# The Hemoprotein Hhy1 Promotes Heme‐Dependent Catalase Activity of Ctt1

**DOI:** 10.1111/mmi.70062

**Published:** 2026-03-08

**Authors:** Tobias Vahsen, Samuel Plante, Berthy Mbuya, Simon Labbé

**Affiliations:** ^1^ Département de Biochimie et de Génomique Fonctionnelle, Faculté de Médecine et Des Sciences de la santé Université de Sherbrooke Sherbrooke Quebec Canada

**Keywords:** catalase, fission yeast, heme, hemoprotein, iron‐regulated gene, protein–protein interaction

## Abstract

In this study, we generated a *Schizosaccharomyces pombe* strain deficient in heme and siderophore biosynthesis, as well as in reductive iron uptake. Using this *hem1*Δ *sib1*Δ *sib2*Δ *fio1*Δ *fip1*Δ mutant strain, we identified Hhy1, a novel protein required for optimal growth when cells rely on exogenous hemin as their sole source of heme. The expression of Hhy1 is upregulated under low‐iron conditions and downregulated when iron levels increase. Fluorescence microscopy analysis shows that the Hhy1‐GFP fluorescent signal mirrors *hhy1* ^*+*^ mRNA expression, being primarily detected in the cytoplasm of iron‐starved *hem1*Δ *sib1*Δ *sib2*Δ *fio1*Δ *fip1*Δ cells. Further monitoring of intracellular heme or its analog zinc mesoporphyrin IX revealed that *hem1*Δ *sib1*Δ *sib2*Δ *fio1*Δ *fip1*Δ cells lacking Hhy1 accumulate these molecules more persistently than cells expressing Hhy1. Consistent with a role for Hhy1 in heme homeostasis, *hhy1*Δ cells exhibit reduced heme‐dependent activity of the catalase Ctt1. Coimmunoprecipitation and bimolecular fluorescence complementation experiments show that Hhy1 interacts with Ctt1. Absorbance spectroscopy and hemin‐agarose pull‐down assays demonstrate that Hhy1 binds hemin, with an equilibrium dissociation constant of 1.09 μM. Taken together, these findings indicate that the iron‐regulated cytoplasmic hemoprotein Hhy1 interacts with Ctt1, promoting its full activation against oxidative stress under hemin‐dependent growth conditions.

## Graphical abstract

A *Schizosaccharomyces pombe* strain deficient in heme (*hem1Δ*) and siderophore (Fc) biosynthesis, as well as high‐affinity iron (Fe) uptake (blue), was used to uncover the hemoprotein Hhy1 that promotes the heme‐dependent activity of the catalase Ctt1. In contrast, *hhy1Δ* cells exhibit reduced Ctt1 activity and increased sensitivity to oxidative stress.

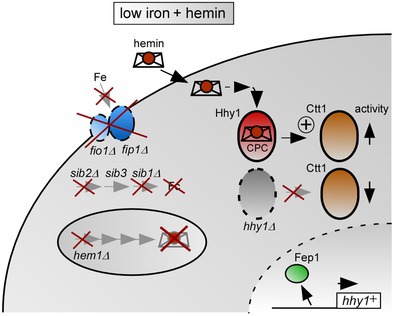

## 1 Introduction

Heme is an iron‐containing protoporphyrin IX macrocyclic molecule that serves as a critical cofactor for several proteins involved in essential biochemical processes such as electron transfer, energy production, xenobiotic detoxification, and free radical scavenging (Donegan et al. 2019; Dutt et al. 2022; Reddi and Hamza 2016; Severance and Hamza 2009; Shimizu et al. 2019; Swenson et al. 2020). In addition, heme serves as a signaling molecule in specific transduction pathways involving kinases and transcription factors (Mense and Zhang 2006; Plante et al. 2025; Shimizu et al. 2019). However, due to its redox‐active nature, imbalanced heme levels can be toxic to cells, as it can generate reactive oxygen species that damage DNA, proteins, and membrane lipids (Kumar and Bandyopadhyay 2005; Severance and Hamza 2009). In addition, heme can be harmful by non‐specifically intercalating into membranes and proteins, thereby interfering with their functions. To counter these effects, organisms have evolved mechanisms to minimize intracellular free heme levels, while preserving its bioavailability.

The heme biosynthesis machinery and heme degradation enzymes play a major role in maintaining proper cellular heme levels (Donegan et al. 2019). These components have traditionally been the primary focus of research on heme homeostasis. Total cellular heme is generally divided into two categories: inert and labile heme. Inert heme represents the major fraction, tightly bound to high‐affinity hemoproteins, and is unavailable for new heme‐requiring enzymatic reactions and processes. In contrast, labile heme is less abundant, exchangeable, and can be utilized by lower‐affinity biomolecules for signaling, trafficking, and cellular stress response reactions. Thus far, however, the properties of labile heme and the identity of the cellular factors involved in its mobilization remain largely uncharacterized (Hanna et al. 2016).

The model organism *Schizosaccharomyces pombe* is a heme prototroph, possessing a highly conserved eight‐step biosynthetic pathway for heme production. Interestingly, *S. pombe* can also acquire heme from external sources, providing it with the unique advantage of utilizing two distinct strategies to fulfill its heme requirements (Labbé et al. 2020). This property of *S. pombe* is reminiscent of the similar ability of *Candida albicans* and *Cryptococcus neoformans* to acquire heme from external sources (Roy et al. 2022; Andrawes et al. 2022; Xue et al. 2022; Bairwa et al. 2020). Since *S. pombe* can take up heme from its environment, most likely from decomposed organic matter derived from decaying fruits, leaves, and plants, it is important to elucidate the mechanisms underlying this process, which may be critical for the colonization of new niches by this microorganism. Leveraging the genetic tractability of *S. pombe*, mutant strains lacking the *hem1* ^+^ gene (*hem1*Δ) have been generated to dissociate the pathways involved in endogenous heme biosynthesis from that of exogenous heme acquisition. Loss of Hem1 blocks the first enzymatic step in the heme biosynthetic pathway, a disruption that is lethal unless the growth medium is supplemented with ‐δ‐aminolevulinate (ALA). Uptake of exogenous ALA by *hem1*Δ cells enables heme biosynthesis to resume at the second enzymatic step, allowing the pathway to proceed through the subsequent reactions required for complete heme production. Alternatively, the viability of *hem1*Δ cells is secured by supplementing them with exogenous hemin (heme chloride) (Mourer et al. 2015; Normant et al. 2018). In this case, *hem1*Δ cells must rely solely on their own heme uptake machinery. Using this *hem1*Δ‐based genetic system in the absence of ALA and in the presence of hemin, studies have identified two independent systems for the assimilation of exogenous hemin (Labbé et al. 2020; Mourer et al. 2015; Normant et al. 2018).

The first system involves the iron‐regulated protein Shu1 (Mourer et al. 2015). Shu1 is a membrane‐bound cell‐surface protein that is sensitive to phosphoinositide‐specific phospholipase activity, which cleaves the glycosylphosphatidylinositol (GPI) anchor from GPI‐anchored proteins. Shu1 contains four Cys residues arranged in a pattern reminiscent of the common fungal extracellular membrane (CFEM) motif (Mourer et al. 2015, 2017). When these cysteines are mutated, Shu1 loses its ability to bind heme compared to the wild‐type protein. When Shu1‐expressing *hem1*Δ cells are grown in the presence of increasing concentrations of exogenous hemin or its analog zinc mesoporphyrin IX (ZnMP), Shu1 undergoes trafficking from the plasma membrane to the vacuolar membrane (Mourer et al. 2019, 2017). Following its uptake mediated by Shu1, the fluorescent ZnMP successively accumulates in the vacuoles and then the cytoplasm. The ABC‐type transporter Abc3 is required to allow the passage of ZnMP from the vacuole to the cytoplasm (Mourer et al. 2017). Hemin‐dependent internalization of Shu1 requires Ubi4‐dependent ubiquitination and the ubiquitin‐conjugating enzyme Ubc13. Furthermore, the intracellular receptor Nbr1 and the Hse1 and Sst6 proteins of the ESCRT (endosomal sorting complex required for transport)‐dependent endosomal sorting system participate in hemin‐dependent vacuolar targeting of Shu1 (Mourer et al. 2019).

The second hemin uptake system counts on the cell‐surface major facilitator transporter Str3 (Normant et al. 2018). Genetic studies using *hem1*Δ *shu1*Δ cells expressing Str3 revealed that their hemin‐dependent growth defect is rescued only when exogenous hemin is supplied at twice the concentration required to overcome the deficiency in *hem1*Δ single mutant cells expressing Shu1 (Normant et al. 2018). Biochemical analyses further revealed that Str3 has a lower affinity for hemin than Shu1, with a *K*_*D*_ of 6.6 μM compared to 2.2 μM for Shu1 (Mourer et al. 2015; Normant et al. 2018). *S. pombe* Str3 is classified as a member of the major facilitator superfamily (MFS) of transporters. Its C‐terminal hydrophilic loop resembles that of a subfamily of fungal MFS‐type siderophore–iron transporters; however, the Str3 C‐terminal extracellular loop is distinct in that it contains two heme‐binding motifs (^530^YX_3_Y^534^ and ^552^SX_4_Y^557^) that interact with hemin. These two motifs may serve as a docking site that facilitates heme transport by Str3 (Normant et al. 2018). Unlike Shu1, Str3 remains localized at the cell surface upon exposure to elevated hemin concentrations (Normant et al. 2021). This observation suggests the involvement of additional cellular components in mobilizing heme following its transport across the plasma membrane by Str3.

In addition to heme uptake, *S. pombe* also utilizes both reductive iron uptake and siderophore‐bound iron transport systems to acquire either inorganic iron or iron‐containing molecules from the environment. For inorganic iron, the multicopper ferroxidase Fio1 forms a complex with the iron permease Fip1, and both proteins are essential for high‐affinity iron uptake at the plasma membrane (Askwith and Kaplan 1997). Regarding siderophores, *S. pombe* synthesizes, accumulates, and excretes ferrichrome (Fc) (Brault et al. 2022; Mercier and Labbé 2010; Plante and Labbé 2019). The biosynthesis of Fc requires the Sib1, Sib2, and Sib3 proteins (Brault et al. 2022; Mbuya et al. 2024; Schrettl et al. 2004). Once secreted into the extracellular environment, Fc‐bound iron can be captured and imported into the cell via the cell‐surface Fc transporter Str1 (Pelletier et al. 2003; Plante and Labbé 2019). To eliminate the contribution of high‐affinity iron uptake and the synthesis of Fc, which binds intracellular and extracellular iron, an *S. pombe* mutant lacking the *hem1* ^+^, *fio1* ^+^, *fip1* ^+^, *sib1* ^+^, and *sib2* ^+^ genes (*hem1*Δ *fio1*Δ *fip1*Δ *sib1*Δ *sib2*Δ) can be used to more specifically investigate the role of the heme uptake system.

In *S. pombe*, several genes encoding proteins involved in heme, iron, and siderophore uptake are regulated at the transcriptional level in response to iron availability by the iron‐responsive GATA‐type transcription factor Fep1 (Pelletier et al. 2002, 2003; Rustici et al. 2007). Under iron‐replete conditions, Fep1 represses the expression of these genes. In contrast, when cells undergo a transition from high to low iron levels, Fep1 becomes inactive and loses its ability to bind GATA regulatory elements on chromatin (Jbel et al. 2009). This inactivation leads to the transcriptional induction of Fep1 target genes, including *shu1* ^+^, *abc3* ^+^, *str3* ^+^, *fio1* ^+^, *fip1* ^+^, *sib1* ^+^, and *sib2* ^+^.

In this study, we found that Hhy1, an uncharacterized protein annotated to contain an amidohydrolase‐like domain, contributes to heme mobilization and catalase activity. We determined that *hhy1* ^+^ expression is repressed under iron‐replete conditions in both *hem1*Δ and *hem1*Δ *sib1*Δ *sib2*Δ *fio1*Δ *fip1*Δ cells. Genetic analyses using *fep1*Δ mutant strains revealed that the iron‐responsive regulator Fep1 contributes to the control of *hhy1* ^+^ expression. Chromatin immunoprecipitation (ChIP) assays consistently showed that TAP‐Fep1 binds to the *hhy1* ^+^ promoter under iron‐replete conditions. Fluorescence microscopy of *hem1*Δ *sib1*Δ *sib2*Δ *fio1*Δ *fip1*Δ *hhy1*Δ cells expressing Hhy1‐GFP revealed that Hhy1 localizes primarily in the cytosol. Functional assays further indicated that Hhy1 is required for maximal hemin‐dependent growth and for optimal induction of Ctt1 catalase activity in *hem1*Δ *sib1*Δ *sib2*Δ *fio1*Δ *fip1*Δ cells. Coimmunoprecipitation and bimolecular fluorescence complementation assays demonstrated an interaction between Hhy1 and Ctt1. Absorbance spectroscopy and hemin‐agarose pull‐down assays further showed that purified Hhy1 binds hemin with a *K*_*D*_ of 1.09 μM. Taken together, these findings identify Hhy1 as a cytosolic hemoprotein that stimulates Ctt1 activity under hemin‐dependent growth conditions.

## 2 Results

### 2.1 The Expression of the *hhy1* ^*+*^ Gene Is Transcriptionally Repressed by Iron in a Fep1‐Dependent Manner in Strains Deficient in Heme Biosynthesis

**FIGURE 1:**
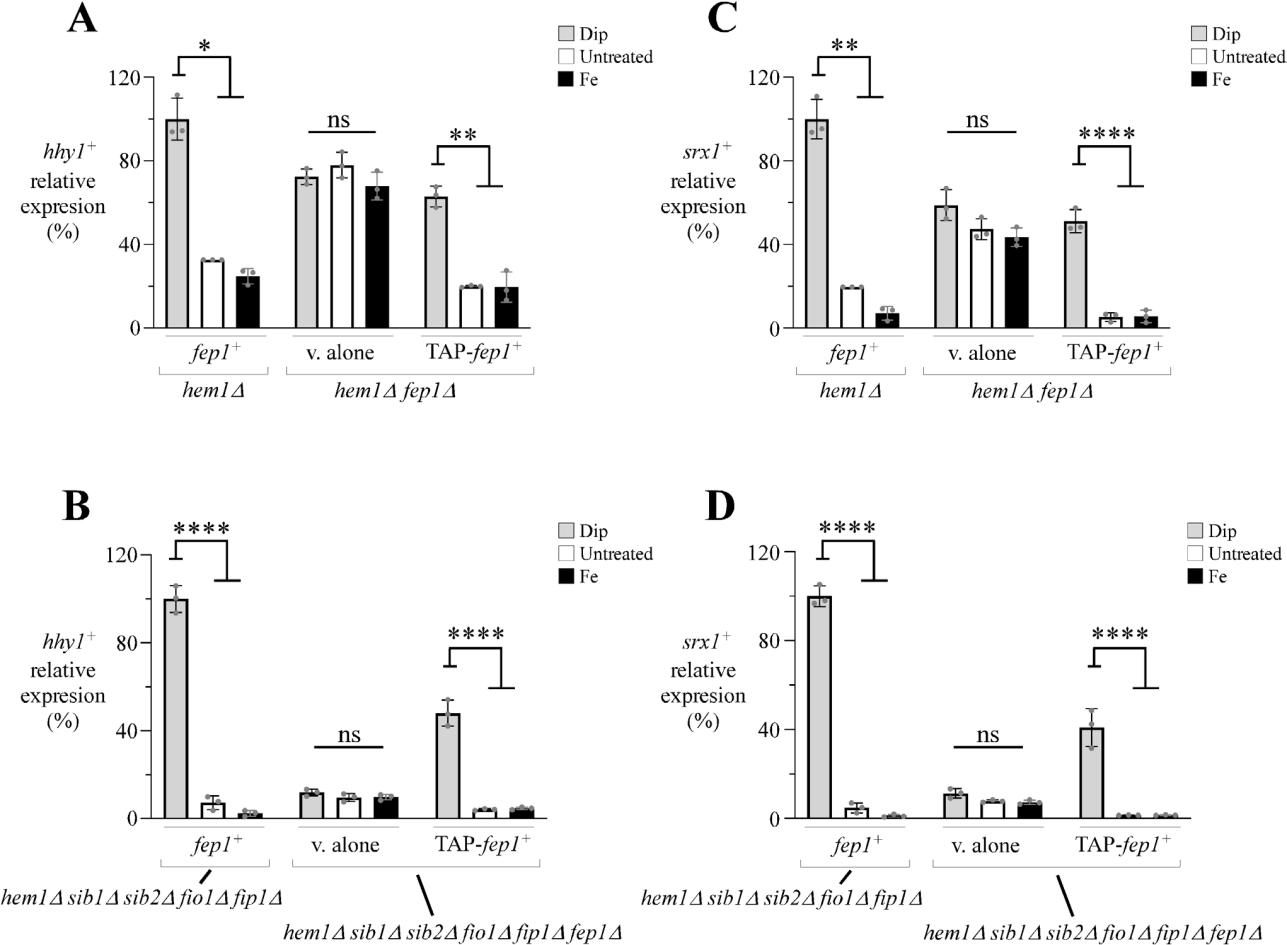
Fep1 plays a role in the repression of *hhy1* ^+^ gene expression in cells lacking heme biosynthesis. (A–D) The indicated strains were precultured in the presence of ALA (200 μM) and FeCl_3_ (50 μM). Cultures were then transferred to an ALA‐free medium containing FeCl_3_ (50 μM) for 5 h. After washing, cultures were transferred to the same medium supplemented with hemin (5 μM) and treated with Dip (300 μM) or FeCl_3_ (Fe, 100 μM), or left untreated for 3 h. Following RNA isolation, the steady‐state mRNA levels of *hhy1* ^+^, *srx1* ^+^, and *act1* ^+^ were analyzed by RT‐qPCR assays. *srx1* ^+^ was included as a control gene known to be repressed by iron, whereas *act1* ^+^ served as an internal control. The graphs represent quantification data from three independent RT‐qPCR experiments (*n* = 3) performed in biological triplicate, with error bars indicating standard deviation (±SD). Asterisks denote statistical significance: *p* < 0.05 (*), *p* < 0.01 (**), and *p* < 0.0001 (****) (two‐way ANOVA with Tuckey's multiple comparisons test against the Dip‐treated strains), whereas ns indicates no significant difference.

On *S. pombe* chromosome I, the *hhy1* ^+^ is located immediately adjacent to *str3* ^+^, followed by *shu1* ^+^. These three genes are co‐oriented in the same direction and arranged sequentially (Rutherford et al. 2024). Given that *shu1* ^+^ and *str3* ^+^ encode proteins involved in heme acquisition and are regulated in response to changes in iron levels (Mourer et al. 2015; Normant et al. 2018), we assessed whether *hhy1* ^+^ expression was similarly regulated by iron availability. To perform these experiments, we used *hem1*Δ mutant cells, which are deficient in endogenous heme biosynthesis and therefore rely on exogenous hemin from the medium as their sole source of heme for growth. In parallel, we employed an isogenic *hem1*Δ *sib1*Δ *sib2*Δ *fio1*Δ *fip1*Δ strain, which is defective in ferrichrome (siderophore) biosynthesis and high‐affinity iron uptake. To determine whether the iron‐responsive transcription factor Fep1 played a role in regulating *hhy1* ^+^, we analyzed *hhy1* ^+^ expression in *hem1*Δ and *hem1*Δ *sib1*Δ *sib2*Δ *fio1*Δ *fip1*Δ strains with or without the *fep1* ^+^ gene. Specifically, *hem1*Δ *fep1*Δ and *hem1*Δ *sib1*Δ *sib2*Δ *fio1*Δ *fip1*Δ *fep1*Δ strains were transformed with either an empty vector (v. alone) or a functional TAP‐*fep1* ^+^ allele. Precultures of *hem1*Δ, *hem1*Δ *fep1*Δ, *hem1*Δ *fep1*Δ expressing *TAP‐fep1* ^+^, *hem1*Δ *sib1*Δ *sib2*Δ *fio1*Δ *fip1*Δ, *hem1*Δ *sib1*Δ *sib2*Δ *fio1*Δ *fip1*Δ *fep1*Δ, and *hem1*Δ *sib1*Δ *sib2*Δ *fio1*Δ *fip1*Δ *fep1*Δ expressing *TAP‐fep1* ^+^ strains were transferred to ALA‐free medium supplemented with FeCl_3_ (50 μM) and incubated for 5 h. At this time point, the ALA‐starved strains were supplemented with hemin (5 μM) and treated with the iron chelator 2,2′‐dipyridyl (Dip, 300 μM), FeCl_3_ (100 μM), or left untreated. After 3 h, total RNA was isolated and analyzed by RT‐qPCR assays. Exposure to Dip resulted in a 3.1‐fold and 13.9‐fold increase in *hhy1* ^+^ mRNA levels compared to the basal levels in the untreated *hem1*Δ and *hem1*Δ *sib1*Δ *sib2*Δ *fio1*Δ *fip1*Δ strains, respectively (Figure 1A,B). In the presence of iron, *hhy1* ^+^ mRNA levels exhibited a decrease relative to the basal levels observed in the same untreated strains (Figure 1A,B). In the absence of Fep1, *hem1*Δ *fep1*Δ cells carrying an empty plasmid exhibited elevated *hhy1* ^+^ expression under both basal and iron‐replete conditions, reaching levels comparable to those observed under iron‐starved conditions (Figure 1A). In the case of *hem1*Δ *sib1*Δ *sib2*Δ *fio1*Δ *fip1*Δ *fep1*Δ cells containing an empty plasmid, the loss of Fep1 did not significantly alter *hhy1* ^+^ expression in response to iron availability. In these cells, *hhy1* ^+^ mRNA levels remained noticeably low across all three tested conditions (Figure 1B). When a functional *TAP‐fep1* ^+^ allele was reintroduced by integration into *hem1*Δ *fep1*Δ and *hem1*Δ *sib1*Δ *sib2*Δ *fio1*Δ *fip1*Δ *fep1*Δ strains, the ability to repress *hhy1* ^+^ gene expression under basal and iron‐replete conditions was restored (Figure 1A,B).

As a control, we concurrently analyzed *srx1* ^+^ transcripts. The expression of *srx1* ^+^ is known to be derepressed under low‐iron conditions and downregulated under both basal and iron‐replete conditions (Vahsen et al. 2023). Consistent with previous findings, *srx1* ^+^ mRNA levels were repressed in both untreated and iron‐treated *hem1*Δ and *hem1*Δ *sib1*Δ *sib2*Δ *fio1*Δ *fip1*Δ strains expressing the endogenous *fep1* ^+^ allele, as well as in the *hem1*Δ *sib1*Δ *sib2*Δ *fio1*Δ *fip1*Δ *fep1*Δ strain expressing the *TAP‐tagged fep1* ^+^ allele (Figure 1C,D). In contrast, in *hem1*Δ *fep1*Δ and *hem1*Δ *sib1*Δ *sib2*Δ *fio1*Δ *fip1*Δ *fep1*Δ strains lacking a functional *fep1* ^+^ allele, *srx1* ^+^ transcript levels remained unchanged and were not significantly affected by iron starvation or repletion (Figure 1C,D). Taken together, these results revealed that *hhy1* ^+^ is an iron‐regulated gene, and that Fep1 plays a role in its repression under iron‐replete conditions.

### 2.2 The *hhy1* ^*+*^ Promoter Is Bound by Fep1 Under Iron‐Replete Conditions

**FIGURE 2:**
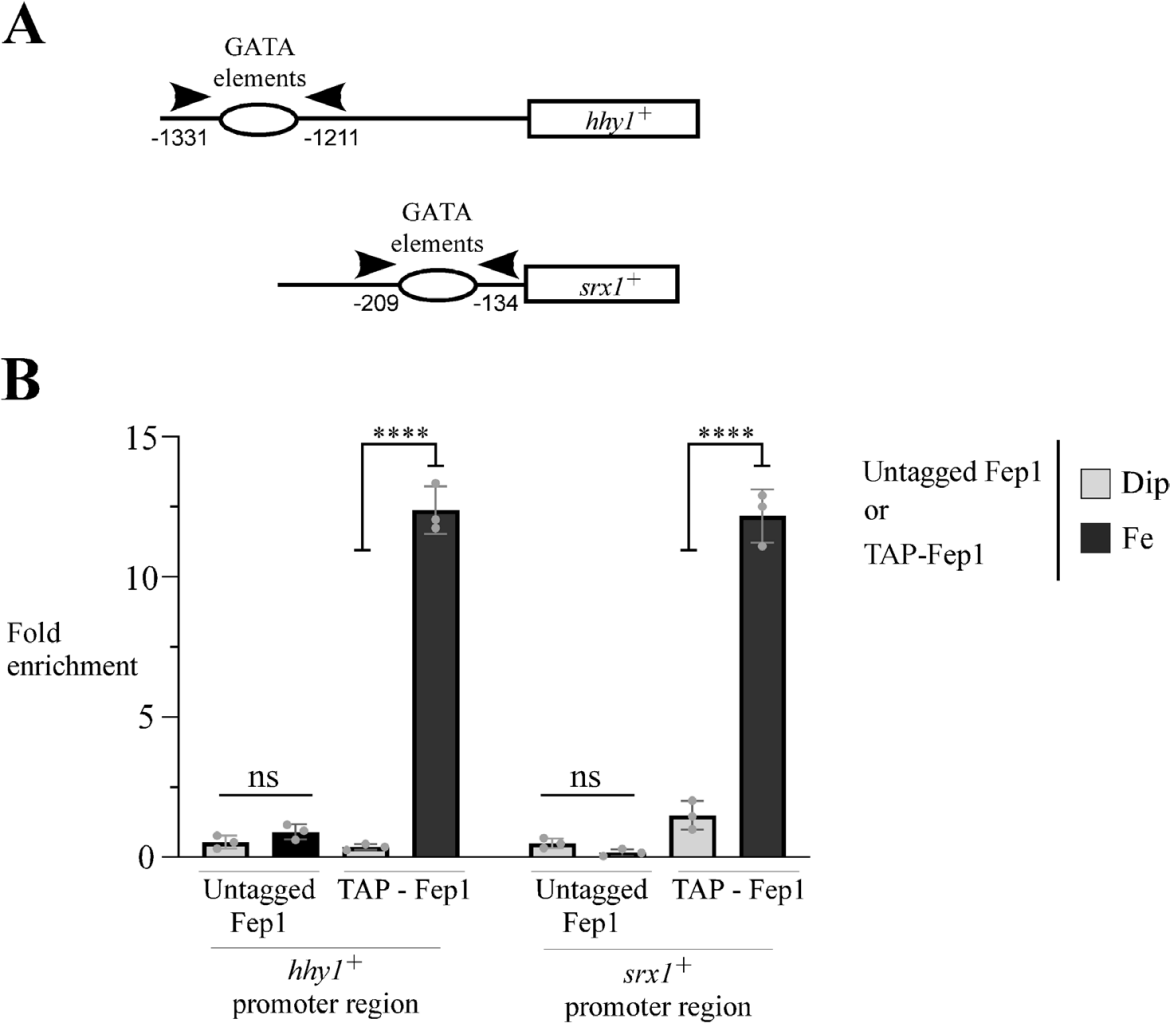
Fep1 is recruited to the *hhy1* ^+^ promoter under iron‐replete conditions. (A) Schematic representations of the *hhy1* ^+^ and *srx1* ^+^ promoter regions indicate the positions of primers used for qPCR analysis. Nucleotide numbers correspond to primer locations relative to the adenine of the initiator codon of each gene. Open ovals indicate promoter regions possessing consensus GATA elements, which are known to be bound by Fep1. (B) Precultures of *hem1*Δ *php4*Δ *fep1*Δ cells expressing either an untagged or TAP‐tagged *fep1* ^+^ allele were washed and transferred to an ALA‐free medium containing FeCl_3_ (50 μM) for 5 h. ALA‐starved cells were then washed and incubated in YES medium supplemented with hemin (5 μM) and treated with Dip (300 μM) or FeCl_3_ (Fe, 100 μM) for 3 h. Cells were subsequently fixed with formaldehyde, lysed, and subjected to chromatin immunoprecipitation using Sepharose‐bound anti‐mouse Ig antibodies to isolate TAP‐Fep1 associated with chromatin. Specific regions of the *hhy1* ^+^ and *srx1* ^+^ promoters were analyzed by qPCR to assess TAP‐Fep1 chromatin occupancy. TAP‐tagged Fep1 density at the promoters was quantified as the enrichment of specific regions relative to an 18S ribosomal DNA coding region. ChIP data are presented as fold enrichment, calculated based on the highest chromatin occupancy measured. Results are shown as averages ± SD from a minimum of three independent experiments (*n* = 3), each conducted in biological triplicate. Asterisks indicate statistical significance (****p < 0.0001, one‐way ANOVA with Dunnett's multiple comparisons test against iron‐replete cells expressing TAP‐Fep1), whereas ns indicates no significant difference.

To further investigate whether Fep1 physically binds the *hhy1* ^+^ promoter under iron‐replete conditions, we used a *hem1*Δ *php4*Δ *fep1*Δ mutant strain expressing either an untagged or a TAP‐tagged *fep1* ^+^ allele. The use of this mutant strain lacking Php4 provided an experimental context in which the iron‐dependent DNA binding activity of Fep1 and TAP‐Fep1 was decoupled from potential changes in their expression levels due to variations in cellular iron status (Mercier and Labbé 2009; Mercier et al. 2008). In this setup, where both *fep1* ^+^ and *TAP‐fep1* ^+^ were constitutively expressed (Mercier et al. 2008), we performed ChIP to determine whether TAP‐Fep1 could bind the *hhy1* ^+^ promoter. This promoter contains GATA elements located between positions −1331 and −1211 relative to the *hhy1* ^+^ initiator codon (Figure 2A). Upon treatment with FeCl_3_, TAP‐Fep1 binding to *hhy1* ^+^ promoter increased 12.4‐fold compared to a control genomic region corresponding to the 18S ribosomal RNA gene (Figure 2B). In contrast, in iron‐starved cells, TAP‐Fep1 was associated with a markedly lower level of immunoprecipitated chromatin at the *hhy1* ^+^ promoter, exhibiting only background levels of occupancy compared to iron‐treated cells (Figure 2B).

As a control, we analyzed the *srx1* ^+^ promoter, which is a known Fep1 target under iron‐replete conditions (Vahsen et al. 2023). As expected, TAP‐Fep1 occupied the *srx1* ^+^ promoter with a 12.2‐fold enrichment relative to the 18S ribosomal DNA control region (Figure 2B). Conversely, in cells treated with Dip, only low levels of the *srx1* ^+^ promoter were immunoprecipitated by TAP‐Fep1 (Figure 2B). Indeed, TAP‐Fep1 showed 8.2‐fold higher occupancy of the *srx1* ^+^ promoter in iron‐replete compared to iron‐starved conditions (Figure 2B). As negative controls, the untagged Fep1 failed to immunoprecipitate significant levels of the *hhy1* ^+^ or *srx1* ^+^ promoter regions (Figure 2B). Taken together, these results showed that TAP‐Fep1 is recruited to the *hhy1* ^+^ promoter under iron‐replete conditions.

### 2.3 Steady‐State Protein Levels and Cellular Localization of Hhy1 Are Negatively Regulated by Iron

**FIGURE 3:**
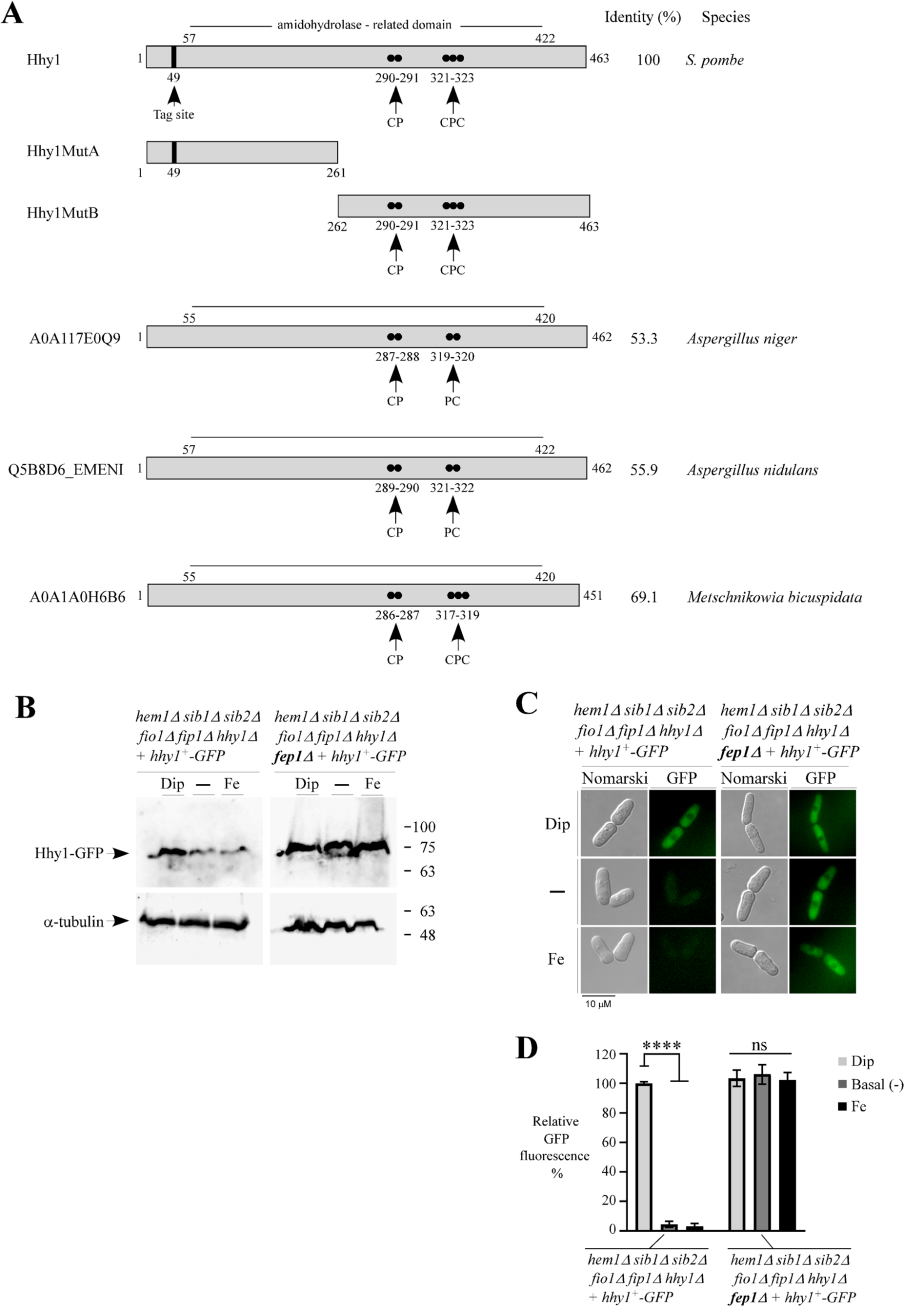
Assessment of Hhy1‐GFP steady‐state protein levels and subcellular localization in response to changes in iron availability. (A) Schematic representations of *S. pombe* Hhy1, two of its mutant derivatives, and three additional Hhy1‐like proteins identified in *Aspergillus niger* (An14g02860), *A. nidulans* (ANIA03194), and *Metschnikowia bicuspidata* are shown. Black dots mark the positions of Cys and Pro residues within predicted CP, CPC, or PC motifs. The site of a short linker inserted in‐frame into Hhy1 for epitope‐tagging is indicated. Amino acid numbers refer to positions relative to the first amino acid residue of each respective protein. (B) *hem1*Δ *sib1*Δ *sib2*Δ *fio1*Δ *fip1*Δ *hhy1*Δ and *hem1*Δ *sib1*Δ *sib2*Δ *fio1*Δ *fip1*Δ *hhy1*Δ *fep1*Δ cells expressing GFP‐tagged Hhy1 were starved for ALA. Following ALA deprivation, cells were supplemented with hemin (5 μM) and treated with Dip (300 μM) or FeCl_3_ (Fe, 100 μM), or left untreated for 3 h. Whole cell extracts were prepared, and aliquots were analyzed by immunoblot assays using anti‐GFP and anti‐α‐tubulin antibodies. Molecular weight markers (in kDa) are indicated on the right. (C) Aliquots of the cultures used in panel (B) were analyzed by fluorescence microscopy to determine the subcellular localization of Hhy1‐GFP. Cell morphology was examined using Nomarski optics. The microscopy results shown are representative of three independent experiments, each performed in biological triplicate. (D) The graph represents quantification of the Hhy1‐GFP fluorescent signal. Data are presented as mean ± SD. Statistical significance is indicated by asterisks: ****p < 0.0001 (two‐way ANOVA with Tuckey's multiple comparisons test against the Dip‐treated *hem1*Δ *sib1*Δ *sib2*Δ *fio1*Δ *fip1*Δ *hhy1*Δ strain expressing GFP‐tagged Hhy1). “ns” indicates no significant difference.

The *hhy1* ^+^ gene encodes a protein consisting of 463 amino acid residues (Figure 3A). Sequence analysis predicts that Hhy1 contains an amidohydrolase‐like domain. However, it remains unclear whether Hhy1 possesses hydrolytic activity or what its potential substrates might be. Although *S. pombe* does not possess any additional Hhy1‐like proteins, searches for putative Hhy1 orthologs in other fungi identified some candidates. These include three Hhy1‐like proteins from *Aspergillus niger*, *Aspergillus nidulans*, and *Mteschnikowia biscuspidata*, which share 53.3%, 55.9%, and 69.1% sequence identity with Hhy1, respectively (Figure 3A). A common feature among these proteins is the presence of a conserved CP, CPC, or inverted CP motif, which is known for its potential to bind heme (Desuzinges‐Mandon et al. 2010; Igarashi et al. 2008; Kuhl et al. 2013; Schubert et al. 2015; Mourer et al. 2017; Shimizu et al. 2019). In the case of the inverted CP motif (Pro‐Cys), it was observed in two of these Hhy1‐like proteins, located at the same position as the CPC motif found in *S. pombe* Hhy1.

To determine whether steady‐state Hhy1 protein levels correlated with *hhy1* ^+^ transcript levels in response to iron and Fep1 availability, we generated *hem1*Δ *sib1*Δ *sib2*Δ *fio1*Δ *fip1*Δ *hhy1*Δ and *hem1*Δ *sib1*Δ *sib2*Δ *fio1*Δ *fip1*Δ *hhy1*Δ *fep1*Δ strains, in which a functional *hhy1* ^+^ *‐GFP* allele was reintroduced by genomic integration. These strains were precultured and transferred to ALA‐free medium supplemented with hemin (5 μM), either left untreated or treated for 3 h with Dip (300 μM) or FeCl_3_ (100 μM). In *hhy1* ^+^ *GFP*‐expressing *hem1*Δ *sib1*Δ *sib2*Δ *fio1*Δ *fip1*Δ *hhy1*Δ cells, Hhy1‐GFP protein levels were predominantly detected under low‐iron conditions (Dip treatment) (Figure 3B). In contrast, Hhy1‐GFP levels were reduced under both basal and high‐iron conditions (Figure 3B). In *hem1*Δ *sib1*Δ *sib2*Δ *fio1*Δ *fip1*Δ *hhy1*Δ *fep1*Δ cells expressing Hhy1‐GFP, protein levels remained consistently high and largely unchanged by the three experimental conditions (Dip, basal, and FeCl_3_) compared to Hhy1GFP‐expressing *hem1*Δ *sib1*Δ *sib2*Δ *fio1*Δ *fip1*Δ *hhy1*Δ cells (Figure 3B).

Concurrent fluorescence microscopy analysis of culture aliquots showed that the Hhy1‐GFP fluorescent signal was primarily localized in the cytoplasm of Hhy1GFP‐expressing *hem1*Δ *sib1*Δ *sib2*Δ *fio1*Δ *fip1*Δ *hhy1*Δ cells under low‐iron conditions (Figure 3C). In contrast, Hhy1‐GFP fluorescence levels were markedly reduced in the same strain grown under basal or high‐iron conditions, exhibiting 95.7% ± 2.1% and 97.0% ± 1.9% decrease, respectively (Figure 3C,D). In *hem1*Δ *sib1*Δ *sib2*Δ *fio1*Δ *fip1*Δ *hhy1*Δ *fep1*Δ cells expressing *hhy1* ^+^ *‐GFP* but lacking *fep1* ^+^, the Hhy1‐GFP fluorescent signal remained consistently elevated, with values of 103.0% ± 5.5% (Dip), 106.0% ± 6.6% (basal), and 102.0% ± 4.9% (iron), regardless of iron availability (Figure 3C,D). Taken together, these results revealed that Hhy1 is a cytoplasmic protein whose expression is upregulated under iron‐limiting conditions or in the absence of Fep1.

### 2.4 Inactivation of the *hhy1* ^*+*^ Gene Results in a Hemin‐Dependent Growth Deficit of *hem1*Δ *sib1*Δ *sib2*Δ *fio1*Δ *fip1*Δ Cells

**FIGURE 4:**
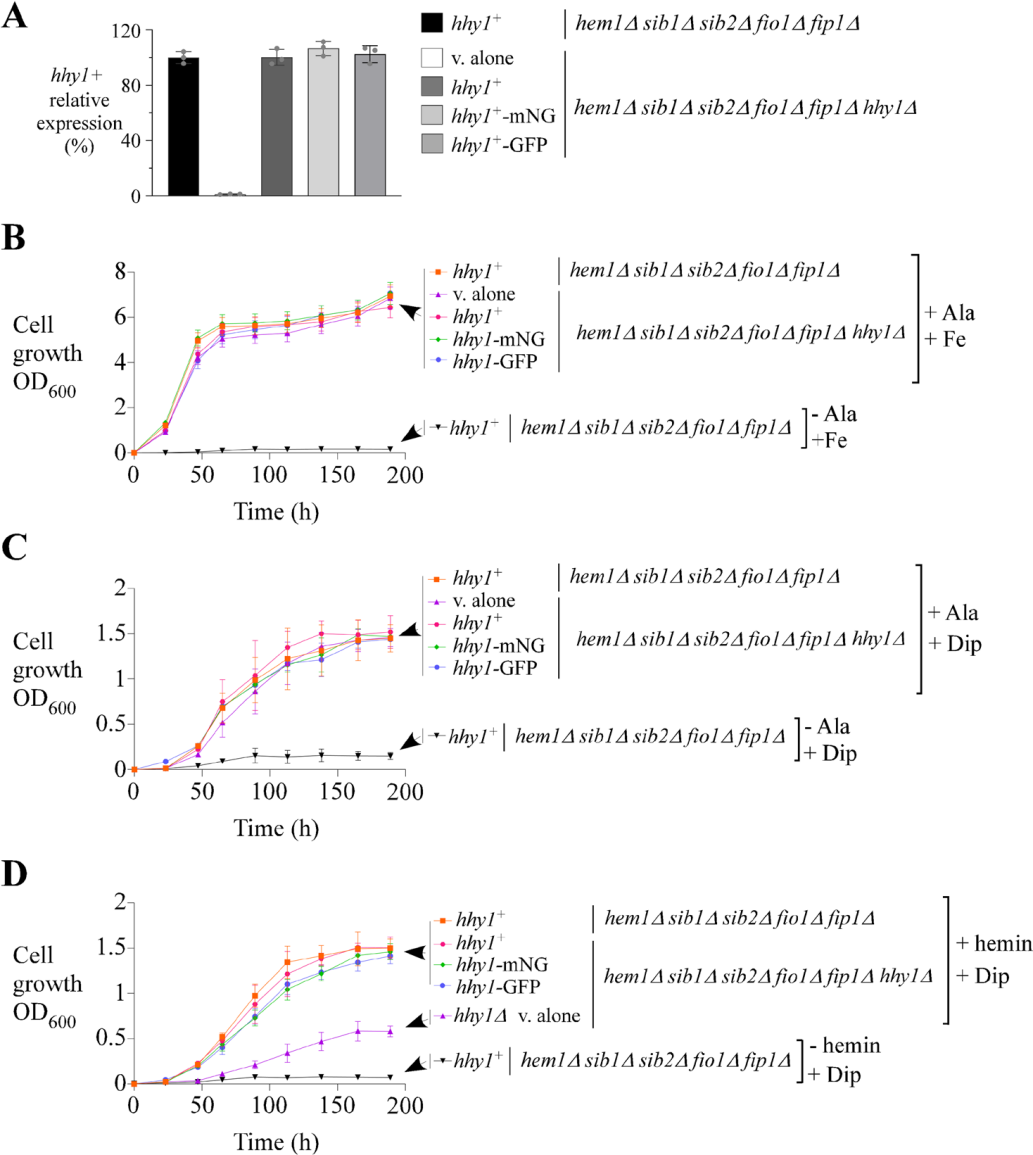
The *hhy1* ^+^ gene is required for maximal hemin‐dependent growth of *hem1*Δ *sib1*Δ *sib2*Δ *fio1*Δ *fip1*Δ cells. (A) The indicated strains were cultured in the presence of ALA (200 μM) and Dip (300 μM). At mid‐logarithmic phase, RNA was extracted from culture aliquots, and *hhy1* ^+^ mRNA levels were analyzed by RT‐qPCR assays. The graph shows quantification from three independent RT‐qPCR experiments (*n* = 3), each performed in biological triplicate. (B–D) The strains from *panel A* were washed, diluted 1000‐fold, and divided into three treatment groups as follows: The presence of ALA (200 μM) and FeCl_3_ (100 μM) (panel B); the presence of ALA and Dip (75 μM) (panel C); and, the presence of hemin (0.25 μM) and Dip (75 μM) (*panel D*). For negative controls, ALA was omitted in the first two treatment groups for *hem1*Δ *sib1*Δ *sib2*Δ *fio1*Δ *fip1*Δ cells expressing *hhy1* ^+^ (panels B and C), whereas hemin was omitted in panel (D) for the same cells. Cell proliferation was monitored under each condition. Strain color codes are as follows: Orange or black, *hem1*Δ *sib1*Δ *sib2*Δ *fio1*Δ *fip1*Δ expressing endogenous *hhy1* ^+^; violet, *hem1*Δ *sib1*Δ *sib2*Δ *fio1*Δ *fip1*Δ *hhy1*Δ containing an empty vector; red, *hem1*Δ *sib1*Δ *sib2*Δ *fio1*Δ *fip1*Δ *hhy1*Δ expressing *hhy1* ^+^; green, *hem1*Δ *sib1*Δ *sib2*Δ *fio1*Δ *fip1*Δ *hhy1*Δ expressing *hhy1* ^+^ *‐mNeonGreen*; and, blue, *hem1*Δ *sib1*Δ *sib2*Δ *fio1*Δ *fip1*Δ *hhy1*Δ expressing *hhy1* ^+^ *‐GFP*. Results represent the mean ± S.D. (error bars) of three independent experiments (*n* = 3).

As previously mentioned, one predicted function of Hhy1 is related to heme homeostasis, as its coding sequence is located adjacent to those of *shu1* ^+^ and *str3* ^+^. Therefore, we investigated whether *hem1*Δ *sib1*Δ *sib2*Δ *fio1*Δ *fip1*Δ *hhy1*Δ cells (lacking Hhy1) exhibit a growth phenotype when relying exclusively on exogenous hemin as their sole source of heme. Prior to conducting cell proliferation assays, the *hem1*Δ *sib1*Δ *sib2*Δ *fio1*Δ *fip1*Δ *hhy1*Δ strain was transformed with integrative plasmids carrying either the *hhy1* ^+^, *hhy1* ^+^ *‐mNeonGreen*, or *hhy1* ^+^ *‐GFP* allele. As a control, the same strain was also transformed with an empty vector (v. alone). These transformed strains were then cultured in medium supplemented with ALA and Dip to assess whether the integrated alleles were transcriptionally expressed at levels comparable to the *hem1*Δ *sib1*Δ *sib2*Δ *fio1*Δ *fip1*Δ strain expressing the endogenous *hhy1* ^+^ allele. The results showed that mRNA levels of the reintegrated *hhy1* ^+^, *hhy1* ^+^ *‐mNeonGreen* and *hhy1* ^+^ *‐GFP* alleles were highly similar among the different strains, except in the *hem1*Δ *sib1*Δ *sib2*Δ *fio1*Δ *fip1*Δ *hhy1*Δ strain transformed with the empty vector, in which *hhy1* ^+^ was absent (Figure 4A).

Once all the above strains reached mid‐logarithmic phase, they were washed, diluted, and divided into three treatment groups. The first group was cultured in medium containing exogenous ALA (200 μM) and FeCl_3_ (100 μM). Under these conditions, all *hem1*Δ‐derived strains, regardless of additional gene disruptions such as *sib1*Δ *sib2*Δ *fio1*Δ *fip1*Δ or *sib1*Δ *sib2*Δ *fio1*Δ *fip1*Δ *hhy1*Δ, and irrespective of whether they expressed untagged *hhy1* ^+^, *hhy1* ^+^ *‐mNeonGreen*, or *hhy1* ^+^ *‐GFP* alleles, exhibited comparable growth patterns. These strains exhibited cell growth to an OD_600_ of 6.0–6.8 after 189 h (Figure 4B). As a negative control, *hem1*Δ *sib1*Δ *sib2*Δ *fio1*Δ *fip1*Δ cells incubated in ALA‐free medium without hemin supplementation failed to grow (Figure 4B,C).

Similar cell proliferation patterns were observed for all strains cultured in medium containing exogenous ALA (200 μM) and Dip (75 μM) (Figure 4C). However, a notable difference was the reduced maximal optical density (OD_600_) under these conditions, which was attributed to the presence of the iron chelator Dip. After 189 h, all strains reached an OD_600_ ranging from 1.4 to 1.5 (Figure 4C). This reduction in growth reflected limited iron availability, which constrained the maximal proliferation of strains lacking *sib1* ^+^, *sib2* ^+^, *fio1* ^+^, and *fip1* ^+^ genes that are involved in iron acquisition (Askwith and Kaplan 1997; Mercier and Labbé 2010; Plante and Labbé 2019).

The last treatment group was cultured in ALA‐free medium supplemented with exogenous hemin (0.25 μM) under low‐iron conditions (75 μM Dip). Under these conditions, the *hem1*Δ *sib1*Δ *sib2*Δ *fio1*Δ *fip1*Δ *hhy1*Δ mutant exhibited poor growth (OD_600_ of 0.51 after 189 h) compared to *hem1*Δ *sib1*Δ *sib2*Δ *fio1*Δ *fip1*Δ cells with an endogenous *hhy1* ^+^ gene (OD_600_ of 1.50 after 189 h) or *hem1*Δ *sib1*Δ *sib2*Δ *fio1*Δ *fip1*Δ *hhy1*Δ cells in which untagged, mNeonGreen‐tagged, or GFP‐tagged *hhy1* ^+^ alleles were re‐integrated (OD_600_ ranging from 1.42 to 1.51 after 189 h) (Figure 4D). As a control, *hem1*Δ *sib1*Δ *sib2*Δ *fio1*Δ *fip1*Δ cells showed no significant growth in ALA‐free medium lacking exogenous hemin under low‐iron conditions (Figure 4D). Taken together, these results revealed that Hhy1 is required to enable maximal growth of heme synthesis‐deficient *hem1*Δ *sib1*Δ *sib2*Δ *fio1*Δ *fip1*Δ cells in ALA‐free medium supplemented with hemin as the sole source of heme.

### 2.5 Disruption of the *hhy1* ^*+*^ Gene in *hem1*Δ *sib1*Δ *sib2*Δ *fio1*Δ *fip1*Δ Cells Results in ZnMP or Heme Persistence

**FIGURE 5:**
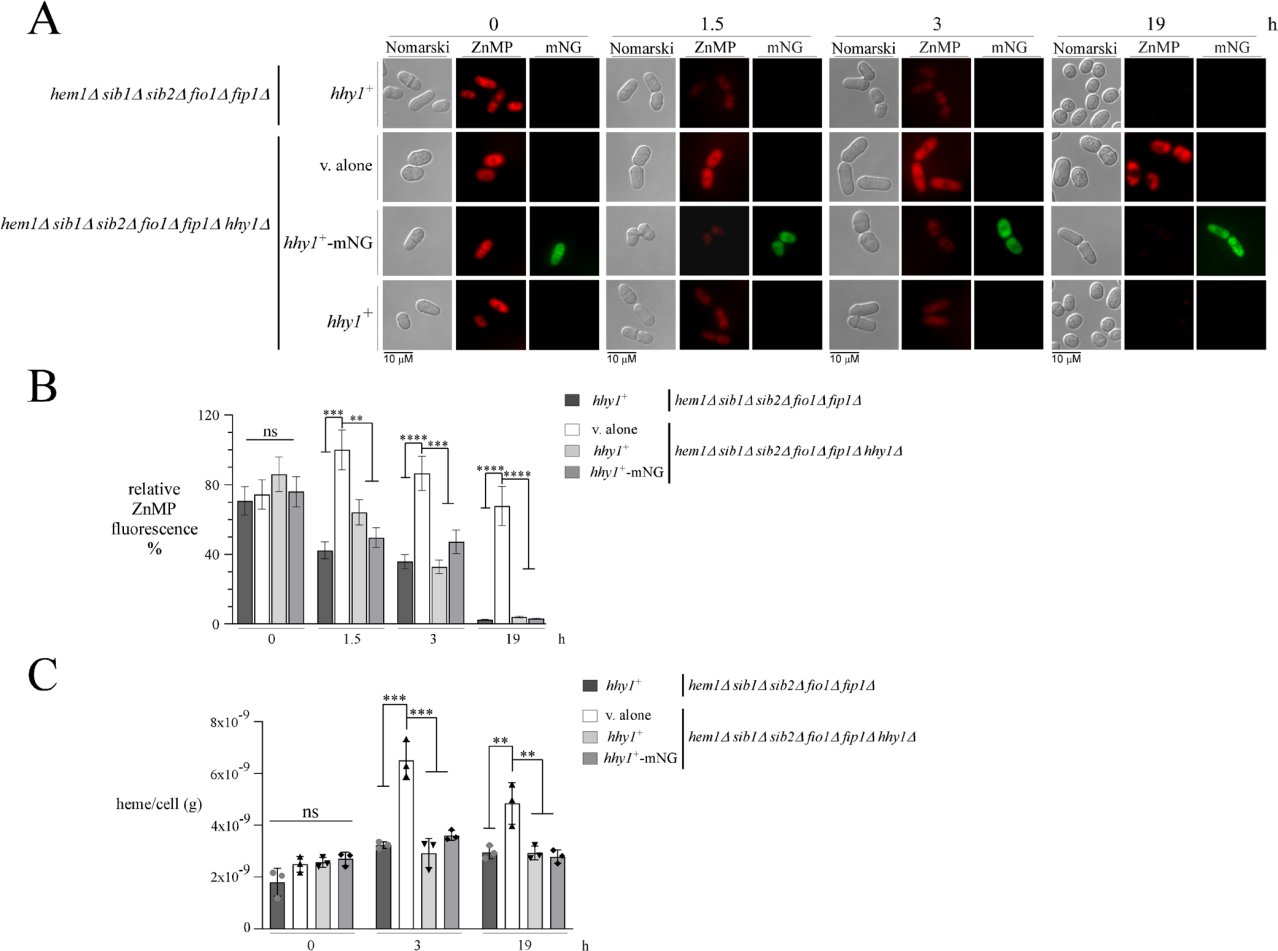
The noniron metalloporphyrin ZnMP and heme persist within *hem1*Δ *sib1*Δ *sib2*Δ *fio1*Δ *fip1*Δ cells lacking Hhy1. (A) The indicated strains were precultured in the presence of ALA, then washed and transferred to an ALA‐free medium containing Dip (25 μM) and hemin (1 μM) for 3 h. ZnMP (10 μM) was added in the final 1.5 h of incubation. After this incubation period, cultures were washed and transferred to a fresh ALA‐free medium containing Dip (25 μM). Fluorescence microscopy was used to detect ZnMP fluorescence at 0, 1.5, 3, and 19 h post‐transfer. Subcellular localization of Hhy1‐mNeonGreen was assessed in *hem1*Δ *sib1*Δ *sib2*Δ *fio1*Δ *fip1*Δ *hhy1*Δ cells expressing the *hhy1* ^+^ *‐mNG* allele. (B) The graph represents quantification of fluorescent ZnMP persistence. The microscopy results are representative of three independent experiments, each performed in biological triplicate. (C) The indicated strains were precultured as described in panel (A), washed, and then transferred to an ALA‐free medium containing Dip (25 μM) for 1.5 h. The cultures were then divided into two groups, either left untreated or supplemented with hemin (0.25 μM). At this point (zero time) and after 3 and 19 h, aliquots of cells were washed, and the total cellular heme content was determined using a fluorometric‐based assay. Results are representative of three independent experiments (*n* = 3). Data are presented as mean ± SD. Statistical significance is indicated by asterisks: **p < 0.01, ***p < 0.001, and ****p < 0.0001 (determined by one‐way ANOVA with Dunnett's multiple comparisons test, using *hem1*Δ *sib1*Δ *sib2*Δ *fio1*Δ *fip1*Δ *hhy1*Δ cells with an empty vector as the reference).

To further investigate the function of Hhy1, we used *hem1*Δ *sib1*Δ *sib2*Δ *fio1*Δ *fip1*Δ cells expressing the endogenous *hhy1* ^+^ allele, as well as *hem1*Δ *sib1*Δ *sib2*Δ *fio1*Δ *fip1*Δ *hhy1*Δ cells carrying either an empty plasmid or integrated plasmids expressing untagged *hhy1* ^+^ or mNeonGreen‐tagged *hhy1* ^+^ alleles. These cells were grown in ALA‐free medium supplemented with hemin (1 μM) under low‐iron conditions. During the final 1.5 h of growth, the heme analog ZnMP (10 μM) was added, after which cells were transferred to an ALA‐free medium containing Dip (25 μM). After 1.5 and 3 h, the ZnMP fluorescent signal decreased significantly in *hem1*Δ *sib1*Δ *sib2*Δ *fio1*Δ *fip1*Δ cells expressing endogenous *hhy1* ^+^ (by 57.7% and 50.6%, respectively) and in *hem1*Δ *sib1*Δ *sib2*Δ *fio1*Δ *fip1*Δ *hhy1*Δ cells expressing either *hhy1* ^+^ (40.0% and 53.8% decrease) or the mNeonGreen‐tagged *hhy1* ^+^ allele (50.3% and 40.0% decrease), compared to cells lacking *hhy1* ^+^ (v. alone) (Figure 5A,B). Furthermore, the ZnMP fluorescent signal persisted in cells lacking Hhy1 after 19 h, in contrast to its disappearance in Hhy1‐ and Hhy1mNeonGreen‐expressing *hem1*Δ *sib1*Δ *sib2*Δ *fio1*Δ *fip1*Δ *hhy1*Δ cells (Figure 5A,B).

To further confirm that the loss of Hhy1 led to cellular heme persistence, all the above‐mentioned strains were cultured under the same conditions, with the exception that 0.25 μM hemin was added instead of ZnMP for 0, 3, and 19 h. At each time point, total cellular heme content was measured using a fluorometric‐based assay (Hanna et al. 2018; Hans et al. 2001; Michener et al. 2012; Sassa 1976). The results showed that *hem1*Δ *sib1*Δ *sib2*Δ *fio1*Δ *fip1*Δ *hhy1*Δ cells harboring an empty plasmid exhibited higher heme levels, with 6.51 ng and 4.84 ng per cell after 3 and 19 h, respectively, compared to *hem1*Δ *sib1*Δ *sib2*Δ *fio1*Δ *fip1*Δ cells expressing endogenous *hhy1* ^+^ (3.23 and 2.95 ng per cell), or *hem1*Δ *sib1*Δ *sib2*Δ *fio1*Δ *fip1*Δ *hhy1*Δ cells expressing either a *hhy1* ^+^ allele (2.92 and 2.93 ng per cell) or an mNeonGreen‐tagged *hhy1* ^+^ allele (3.60 and 2.79 ng per cell) at the same time points (Figure 5C). Together, these results indicated that cells deficient in heme biosynthesis and lacking Hhy1 exhibit persistent detection of heme when cultured in ALA‐free medium supplemented with hemin.

### 2.6 Hhy1 Is a Hemin‐Binding Protein

**FIGURE 6:**
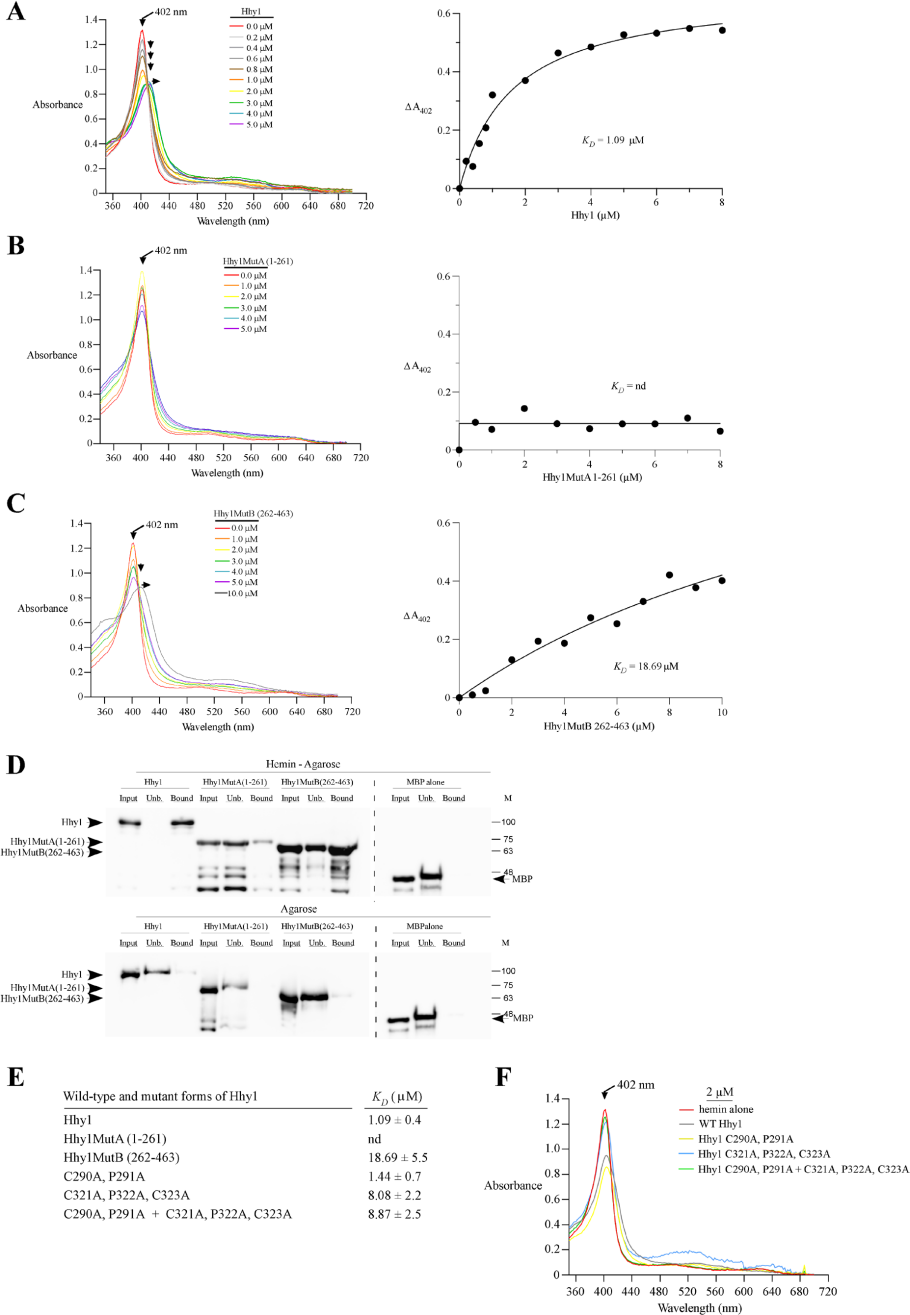
Hhy1 binds to hemin. (A–C) Wild‐type Hhy1 (panel A), Hhy1MutA (panel B), and Hhy1MutB (panel C) were expressed in *E. coli* , purified, and analyzed by differential spectral titration using 5 μM hemin. Titration assays (left panels) were performed with increasing concentrations of the indicated proteins (ranging from 0 to 5 μM or 0–10 μM). Protein concentrations are color‐coded as follows: Red, 0 μM; light gray, 0.2 μM; gray, 0.4 μM; dark gray, 0.6 μM; brown, 0.8 μM; orange, 1.0 μM; yellow, 2.0 μM; green, 3.0 μM; blue, 4.0 μM; violet, 5.0 μM; and, black, 10.0 μM. The right panels display the hemin‐binding curves for Hhy1, Hhy1MutA, and Hhy1MutB, generated by plotting changes in absorbance at the Soret peak as a function of the indicated protein concentrations. Hhy1 and Hhy1MutB bound hemin with *K*_*D*_ values of 1.09 × 10^−6^ M and 18.69 × 10^−6^ M, respectively. In contrast, Hhy1MutA showed no detectable significant interaction with hemin, precluding *K*_*D*_ determination. (D) Aliquots of the purified proteins from panels (A–C) were incubated with either hemin‐agarose or control agarose beads. The presence of the indicated protein in the flow‐through (unbound fraction, Unb.) and the bound fraction was assessed by immunoblotting using an anti‐MBP antibody. As a negative control, purified MBP alone was only detected in the unbound fraction of the hemin‐agarose beads. (E) Additional Hhy1 mutant derivatives were bacterially expressed, purified, and subjected to spectroscopic analysis. For each mutant, the *K*_*D*_ ± SD was determined to evaluate whether specific amino acid residues, particularly those in putative CP and CPC motifs, are required for optimal hemin binding by Hhy1. (F) Example of spectral titration analysis of the wild‐type protein and CP mutant forms of Hhy1 using 2 μM of the indicated proteins with the following color code: Red, hemin alone; gray, wild‐type Hhy1; yellow, Hhy1‐C290A/P291A; blue, Hhy1‐C321A/P322A/C323A; and, green, Hhy1‐C290A/P291A/C321A/P322A/C323A.

Given that Hhy1 is required for the optimal utilization of exogenous hemin when it serves as the sole source of heme, we examined its ability to bind hemin. To assess this, wild‐type Hhy1 and two mutated versions were expressed in *E. coli* . One mutant, denoted Hhy1MutA, comprised the first 261 amino acids of the protein, whereas the second, Hhy1MutB, consisted of residues 262–463. The MBP‐tagged wild‐type and mutant forms of Hhy1 were purified using amylose affinity chromatography. Prior analyzing the purified proteins by spectroscopy, we validated that a control reaction containing hemin but lacking Hhy1 displayed an absorption peak corresponding to the Soret peak at 402 nm (Figure 6A). Upon addition of increasing concentrations of wild‐type Hhy1 to a fixed concentration of hemin, we observed a decrease in absorbance and a detectable red shift in the Soret peak (Figure 6A). Although to a lesser extent than the wild‐type protein, addition of Hhy1MutB also led to a reduction of the Soret peak, along with a delayed red shift in hemin absorbance at higher protein concentrations (Figure 6A–C). Analysis of the absorbance changes as a function of protein concentrations indicated that wild‐type Hhy1 and Hhy1MutB bound hemin with *K*_*D*_ values of 1.09 × 10^−6^ M and 18.69 × 10^−6^ M, respectively. In contrast, Hhy1MutA did not produce consistent changes of the Soret peak across increasing protein concentrations, preventing determination of its *K*_*D*_ value (Figure 6B).

To further characterize the heme‐binding ability of the wild‐type and mutant forms of Hhy1, we carried out pull‐down assays using either hemin‐conjugated or unconjugated agarose beads. The wild‐type Hhy1 protein was retained on the hemin‐agarose beads (Figure 6D, bound) and was absent from the flow‐through (unbound) fraction (Figure 6D, Unb). For Hhy1MutB, the protein was detected in both the bound and unbound fractions, with a higher amount in the bound fraction (Figure 6D). In contrast, the Hhy1MutA mutant was primarily found in the unbound fraction, with only trace amounts in the bound fraction, indicating a drastic loss of hemin‐binding affinity (Figure 6D). As controls, wild‐type Hhy1 and its mutant forms were detected primarily in the unbound fractions when unconjugated agarose beads were used in pull‐down experiments. Furthermore, purified MBP protein was detected only in the unbound fraction, regardless of whether the pull‐down assays were performed with hemin‐conjugated or unconjugated agarose beads (Figure 6D).

Because the C‐terminal region from amino acids 262–463 of Hhy1 exhibited the ability to bind hemin, albeit with lower affinity than the full‐length protein, we sought to identify specific amino acid residues within this region that contribute to hemin interaction. Several hemoproteins contain a conserved CP motif known to play a role in heme binding (Shimizu et al. 2019). Notably, one CP motif (Cys^290^‐Pro^291^) and one CPC motif (Cys^321^‐Pro^322^‐Cys^323^) were identified within the C‐terminal region of Hhy1. To assess the role of these motifs in hemin binding, we generated mutants in which the Cys and Pro residues were substituted with alanine residues in one or both motifs within the context of the full‐length protein. Following bacterial expression and purification of these mutated proteins, spectroscopic analysis revealed that the Hhy1‐C290A/P291A mutant bound hemin with a *K*_*D*_ value of 1.44 × 10^−6^ M, similar to that of wild‐type Hhy1 (1.09 × 10^−6^ M), indicating that the first CP motif (Cys^290^‐Pro^291^) was dispensable for hemin binding (Figure 6E). In contrast, the Hhy1‐C321A/P322A/C323A mutant exhibited a significantly reduced binding affinity for hemin, with a *K*_*D*_ value of 8.08 × 10^−6^ M, which is 7.4‐fold lower than that of the wild‐type Hhy1 (Figure 6E). In the case of the double mutant Hhy1‐C290A/P291A/C321A/P322A/C323A, results showed that it bound hemin with a *K*_*D*_ value of 8.87 × 10^−6^ M, comparable to that of the CPC motif mutant alone (Hhy1‐C321A/P322A/C323A) (Figure 6E). Spectroscopic analysis using 2 μM of wild‐type and CP mutant forms of Hhy1 revealed a decrease in absorbance and a red shift in the Soret peak for the wild‐type protein and the Hhy1‐C290A/P291A mutant (Figure 6F). In contrast, the Hhy1‐C321A/P322A/C323A and Hhy1‐C290A/P291A/C321A/P322A/C323A mutants did not exhibit significant changes in the Soret peak (Figure 6F). Taken together, these results indicated that Hhy1 interacts directly with hemin, and that the CPC motif within its C‐terminal region is required for optimal high‐affinity binding with hemin.

### 2.7 Hhy1 Contributes to the Full Activation of the Catalase Ctt1

**FIGURE 7:**
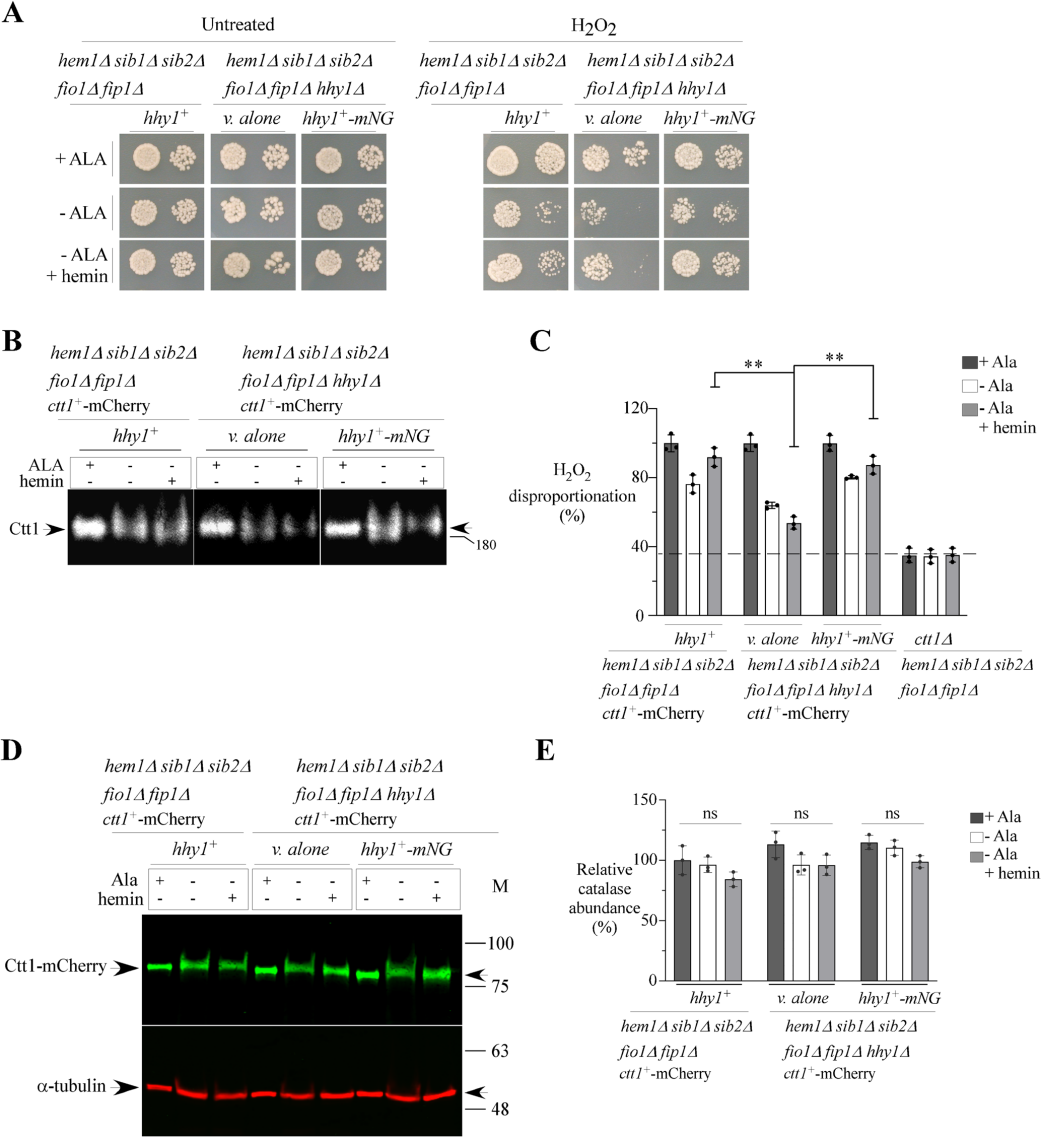
Production of fully active Ctt1 requires Hhy1. (A) The indicated strains were precultured in YES medium containing ALA (25 μM) and FeCl_3_ (25 μM). At mid‐logarithmic phase, the cultures were washed, resuspended in YES medium supplemented with Dip (25 μM), and then divided into two treatment groups: one with ALA (100 μM) and another one without ALA, both incubated for 6 h. In the case of cultures without ALA, hemin (1 μM) was added to half of the samples during the final 1.5 h of incubation, thus forming a third treatment group. After incubation, cultures were serially diluted (500 cells/10 μL and 50 cells/10 μL) and spotted onto YES medium either containing ALA alone or supplemented with H_2_O_2_ (0.2 mM). (B) Total protein extracts from cultures treated as described in *panel A* were separated by non‐denaturing polyacrylamide gel electrophoresis. Catalase activity was assessed and visualized by identifying H_2_O_2_‐cleared bands following potassium ferricyanide/ferric chloride staining. (C) Aliquots of cell lysates from *panel B* were analyzed spectrophotometrically at 240 nm to measure catalase activity. Activity was calculated based on the rate of H_2_O_2_ decomposition, proportional to the reduction in absorbance at 240 nm. Data are presented as mean ± SD. Statistical significance is indicated by asterisks: **p < 0.01 (determined by one‐way ANOVA with Dunnett's multiple comparisons test, using *hem1*Δ *sib1*Δ *sib2*Δ *fio1*Δ *fip1*Δ *ctt1* ^+^ *‐mCherry* cells expressing endogenous *hhy1* ^+^ as the reference). (D) Total protein extracts from each treatment group of cultures were analyzed by immunoblotting using anti‐Cherry and anti‐α‐tubulin antibodies. (E) Quantification of results from three independent experiments (*n* = 3), including the western blot data shown in panel (D). Histogram values represent the mean ± SD. The symbol “ns” stands for non‐significant difference.

Our results revealed an imbalance in intracellular heme levels in *hem1*Δ *sib1*Δ *sib2*Δ *fio1*Δ *fip1*Δ cells lacking Hhy1 under heme‐dependent growth conditions (Figure 5). Since a number of heme‐dependent proteins, such as the catalase Ctt1, help protect cells against oxidative stress, we investigated whether the loss of Hhy1 increased cellular sensitivity to oxidative stress. *hem1*Δ *sib1*Δ *sib2*Δ *fio1*Δ *fip1*Δ cells expressing an endogenous *hhy1* ^+^ allele, as well as *hem1*Δ *sib1*Δ *sib2*Δ *fio1*Δ *fip1*Δ *hhy1*Δ cells carrying either an empty vector or a functional *mNeonGreen‐tagged hhy1* ^+^ allele, were precultured and then divided into three treatment groups. In the first two groups, cells were incubated in the presence of Dip (25 μM) either with or without ALA supplementation. In the case of cultures without ALA, hemin (1 μM) was added to half of the samples, thus forming a third treatment group. After 6 h of incubation, cultures were spotted on ALA‐supplemented media containing either 0 or 0.2 mM H_2_O_2_. As shown in Figure 7A, *hem1*Δ *sib1*Δ *sib2*Δ *fio1*Δ *fip1*Δ *hhy1*Δ cells exhibited a growth defect on H_2_O_2_‐containing medium compared to both *hem1*Δ *sib1*Δ *sib2*Δ *fio1*Δ *fip1*Δ cells expressing *hhy1* ^+^ and *hem1*Δ *sib1*Δ *sib2*Δ *fio1*Δ *fip1*Δ *hhy1*Δ cells expressing *mNeonGreen‐tagged hhy1* ^+^ (Figure 7A). These results showed that loss of Hhy1 function in *hem1*Δ *sib1*Δ *sib2*Δ *fip1*Δ *fio1*Δ cells leads to growth defect under H_2_O_2_‐mediated oxidative stress conditions.

Based on this phenotype, we next examined catalase activity in cell extracts from *hem1*Δ *sib1*Δ *sib2*Δ *fio1*Δ *fip1*Δ cells coexpressing *ctt1* ^+^ *‐mCherry* and *hhy1* ^+^, compared to *hem1*Δ *sib1*Δ *sib2*Δ *fio1*Δ *fip1*Δ *hhy1*Δ *ctt1* ^+^ *‐mCherry* cells, under the previously described growth conditions. In these strains, the *mCherry* coding sequence was integrated downstream and in‐frame with the 3′ end of *ctt1* ^+^. Using in‐gel activity assays with potassium ferricyanide/ferric chloride staining (Lorusso et al. 2022; Pezzoni et al. 2018), results showed that catalase activity was highest under ALA‐replete conditions, regardless of the presence or absence of Hhy1 (Figure 7B,C). In contrast, under ALA‐depleted conditions, without or with hemin supplementation (1 μM), catalase activity was reduced in extracts from *hem1*Δ *sib1*Δ *sib2*Δ *fio1*Δ *fip1*Δ *hhy1*Δ *ctt1* ^+^ *‐mCherry* cells carrying an empty vector, compared to those expressing endogenous Hhy1 or Hhy1‐mNeonGreen (Figure 7B,C). Parallel spectrophotometric liquid catalase assays confirmed these findings: catalase activity in *hem1*Δ *sib1*Δ *sib2*Δ *fio1*Δ *fip1*Δ *hhy1*Δ *ctt1* ^+^ *‐mCherry* cells was reduced by 12.4% ± 1.8% and 39.2% ± 3.6%, respectively, compared to *hem1*Δ *sib1*Δ *sib2*Δ *fio1*Δ *fip1*Δ *ctt1* ^+^ *‐mCherry* cells expressing *hhy1* ^+^ when grown in ALA‐free medium supplemented with Dip or Dip plus hemin (Figure 7C). Similarly, catalase activity was 16.3% ± 1.7% and 34.6% ± 3.5% lower in extracts from *hem1*Δ *sib1*Δ *sib2*Δ *fio1*Δ *fip1*Δ *hhy1*Δ *ctt1* ^+^ *‐mCherry* cells compared to those expressing Hhy1‐mNeonGreen under the same conditions (Figure 7C). To assess H_2_O_2_ breakdown independently of Ctt1 activity, we performed liquid catalase assays using *hem1*Δ *sib1*Δ *sib2*Δ *fio1*Δ *fip1*Δ *ctt1*Δ cells grown under the same three treatment conditions (Figure 7C). In the absence of Ctt1, the H_2_O_2_ decomposition was consistently lower. For instance, under ALA‐depleted conditions with Dip and hemin, catalase activity was reduced by 57.8% ± 3.9% in *hem1*Δ *sib1*Δ *sib2*Δ *fio1*Δ *fip1*Δ *ctt1*Δ extracts compared to those from *hem1*Δ *sib1*Δ *sib2*Δ *fio1*Δ *fip1*Δ *ctt1* ^+^ *‐mCherry* cells. Furthermore, the residual H_2_O_2_ decomposition observed in the absence of Ctt1 remained constant across all conditions tested, indicating that the reduction in catalase activity caused by Hhy1 deletion was primarily attributable to Ctt1.

To verify the presence of the Ctt1‐mCherry protein in *hem1*Δ *sib1*Δ *sib2*Δ *fio1*Δ *fip1*Δ *hhy1* ^+^ *ctt1* ^+^ *‐mCherry* cells, as well as in *hem1*Δ *sib1*Δ *sib2*Δ *fio1*Δ *fip1*Δ *hhy1*Δ *ctt1* ^+^ *‐mCherry* cells carrying either an empty vector or the *hhy1* ^+^ *‐mNeonGreen* allele, whole‐cell extracts were prepared and analyzed by immunoblotting. The results showed detectable and comparable levels of Ctt1 in the strain lacking Hhy1 (*hhy1*Δ), indicating that the reduced catalase activity observed in *hem1*Δ *sib1*Δ *sib2*Δ *fio1*Δ *fip1*Δ *hhy1*Δ *ctt1* ^+^ *‐mCherry* cells was not due to impaired Ctt1 expression (Figure 7D,E). Taken together, these findings showed that Hhy1 contributes to Ctt1 activation when ALA‐starved cells are grown under low‐iron conditions, with or without hemin supplementation.

### 2.8 Hhy1‐GFP and Ctt1‐mCherry Are Interacting Partners

**FIGURE 8:**
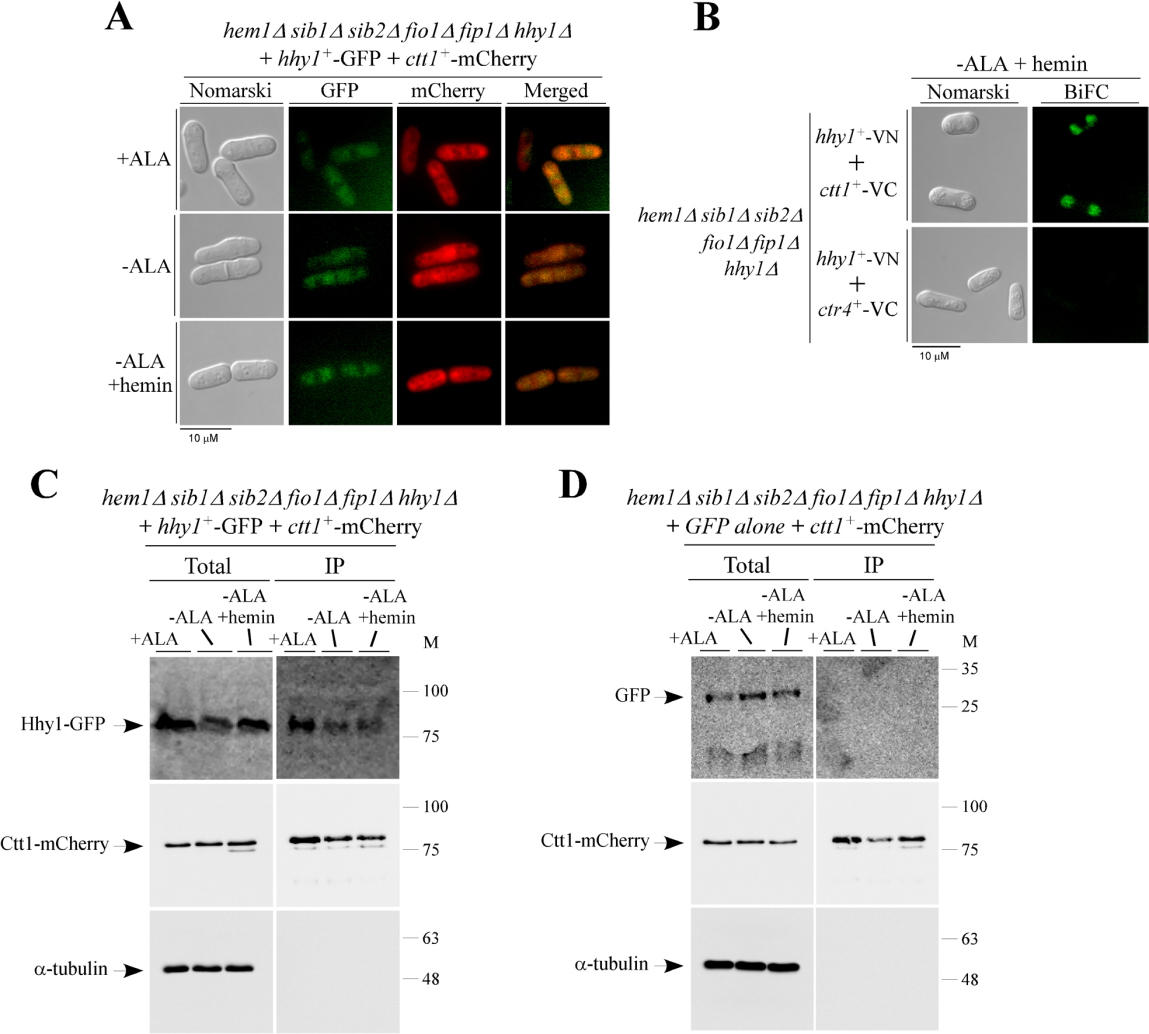
Hhy1‐GFP and Ctt1‐mCherry colocalize and interact with one another in *S. pombe* cells. (A) *hem1*Δ *sib1*Δ *sib2*Δ *fio1*Δ *fip1*Δ *hhy1*Δ cells co‐expressing Hhy1‐GFP and Ctt1‐mCherry were precultured in YES medium supplemented with ALA (25 μM) and FeCl_3_ (25 μM). Cells in mid‐logarithmic phase were washed and transferred to ALA‐free medium supplemented with Dip (25 μM), then either treated with ALA (100 μM) or left untreated for 6 h. In cultures not receiving ALA, hemin (1 μM) was added to half of the samples during the final 1.5 h of incubation. Fluorescence microscopy was conducted to visualize the localization of Hhy1‐GFP (center left) and Ctt1‐mCherry (center right). Merged images of Hhy1‐GFP and Ctt1‐mCherry are shown in the *far‐right panels*. Cell morphology was assessed using Nomarski optics (far left). (B) ALA‐starved *hem1*Δ *sib1*Δ *sib2*Δ *fio1*Δ *fip1*Δ *hhy1*Δ cells coexpressing Hhy1‐VN and Ctt1‐VC or Hhy1‐VN and Ctr4‐VC in the presence of hemin (1 μM) were examined by fluorescence microscopy for BiFC signal detection. White arrowheads indicate cytosolic BiFC signals resulting from Hhy1VN/Ctt1VC interactions. Ctr4, a cell‐surface copper transporter unrelated to Hhy1, served as a negative control. Microscopy results are representative of three independent experiments, each performed in biological triplicate. (C, D) Whole‐cell protein extracts (Total) from the cultures described in panel (A) were subjected to coimmunoprecipitation (IP) using an anti‐mCherry antibody coupled to protein G‐Sepharose beads. As a control, *hem1*Δ *sib1*Δ *sib2*Δ *fio1*Δ *fip1*Δ *hhy1*Δ cells co‐expressing Ctt1‐mCherry with GFP alone were cultured as described in panel (A) and processed in parallel. Total protein extracts from these cells were included in the coimmunoprecipitation assays. Immunoprecipitated fractions (IP) were analyzed by immunoblotting using anti‐GFP, anti‐mCherry, and anti‐α‐tubulin antibodies. Aliquots of the total protein extracts were also probed prior to IP to confirm the expression of epitope‐tagged proteins. Molecular weight markers (kDa) are shown on the right side of each panel for reference.

Given the role of Hhy1 in Ctt1 activation, we examined their subcellular localization when coexpressed in *hem1*Δ *sib1*Δ *sib2*Δ *fio1*Δ *fip1*Δ *hhy1*Δ cells carrying *hhy1* ^+^ *‐GFP* and *ctt1* ^+^ *‐mCherry* alleles. For *ctt1* ^+^, the mCherry coding sequence was directly integrated at its chromosomal locus, whereas for *hhy1* ^+^, the *hhy1* ^+^ *‐GFP* fusion allele was reintroduced by integration into the yeast genome. Cells were precultured in the presence of ALA (25 μM) and FeCl_3_ (25 μM), then transferred to ALA‐free medium supplemented with Dip (25 μM). They were either left untreated or treated with ALA (100 μM) for 6 h. In cultures not receiving ALA, hemin (1 μM) was added to half of the samples during the final 1.5 h of incubation. Green and red fluorescence signals corresponding to Hhy1‐GFP and Ctt1‐mCherry, respectively, were predominantly observed throughout the cytoplasm, regardless of the presence or absence of ALA. Similarly, in ALA‐free medium supplemented with Dip, or with both Dip and hemin, the fluorescence signals remained cytoplasmic and did not exhibit significant changes (Figure 8A). Given their common cytosolic localization, it was conceivable that Hhy1 and Ctt1 may interact directly within this compartment. To investigate this possibility, we employed a BiFC approach by fusing the N‐terminal (VN) and C‐terminal (VC) fragments of Venus to Hhy1 and Ctt1, respectively. To generate Hhy1‐VN, an integrative plasmid carrying the *hhy1* ^+^ *‐VN* allele under the control of its native promoter was transformed into *hem1*Δ *sib1*Δ *sib2*Δ *fio1*Δ *fip1*Δ *hhy1*Δ cells. These transformed cells were then subjected to site‐specific integration of a pFA6A‐VC‐kanMX6 cassette, allowing replacement of the wild‐type allele at either the *ctt1* ^+^ or *ctr4* ^+^ locus with a *VC‐tagged* version. The resulting strains were incubated in ALA‐free medium and subsequently supplemented with hemin (1 μM). Microscopy analysis revealed a cytoplasmic BiFC signal in cells coexpressing Hhy1‐VN and Ctt1‐VC, indicating interaction between the two proteins (Figure 8B). In contrast, no BiFC signal was detected when Hhy1‐VN and Ctr4‐VC were coexpressed as a negative control under the same conditions. This is consistent with the fact that Ctr4, a cell‐surface copper transporter, is functionally unrelated to Hhy1 (Figure 8B) (Ioannoni et al. 2010).

To further investigate the ability of Hhy1 to associate with Ctt1, whole‐cell extracts were prepared from culture aliquots of *hem1*Δ *sib1*Δ *sib2*Δ *fio1*Δ *fip1*Δ *hhy1*Δ cells coexpressing Hhy1‐GFP and Ctt1‐mCherry, under the same three treatment conditions used for their subcellular localization analysis. Following extraction, the cell lysates were incubated with protein G‐Sepharose beads that had been coupled with anti‐mCherry antibodies. Immunoblot analysis of the proteins bound to the beads revealed the presence of both Ctt1‐mCherry and Hhy1‐GFP in the immunoprecipitated (IP) fraction (Figure 8C). While the interaction between Ctt1‐mCherry and Hhy1‐GFP was detected in extracts from cells grown in the presence or absence of ALA, a reduced amount of Hhy1‐GFP was observed in the IP fraction of ALA‐starved cells, whether or not they were supplemented with hemin (Figure 8C). Nonetheless, this Hhy1‐Ctt1 association was specific, as coimmunoprecipitation assays using control *hem1*Δ *sib1*Δ *sib2*Δ *fio1*Δ *fip1*Δ *hhy1*Δ cells coexpressing GFP alone and Ctt1‐mCherry failed to show any interaction between the two proteins (Figure 8D). As an additional control, the specificity of the coimmunoprecipitation was validated by the absence of the unrelated α‐tubulin protein in the IP fractions (Figure 8C,D). Taken together, these results revealed that Hhy1‐GFP and Ctt1‐mCherry specifically interact to form a heteroprotein complex in fission yeast.

### 2.9 Purified Ctt1‐His_6_ and MBP‐Hhy1 Interact In Vitro

**FIGURE 9:**
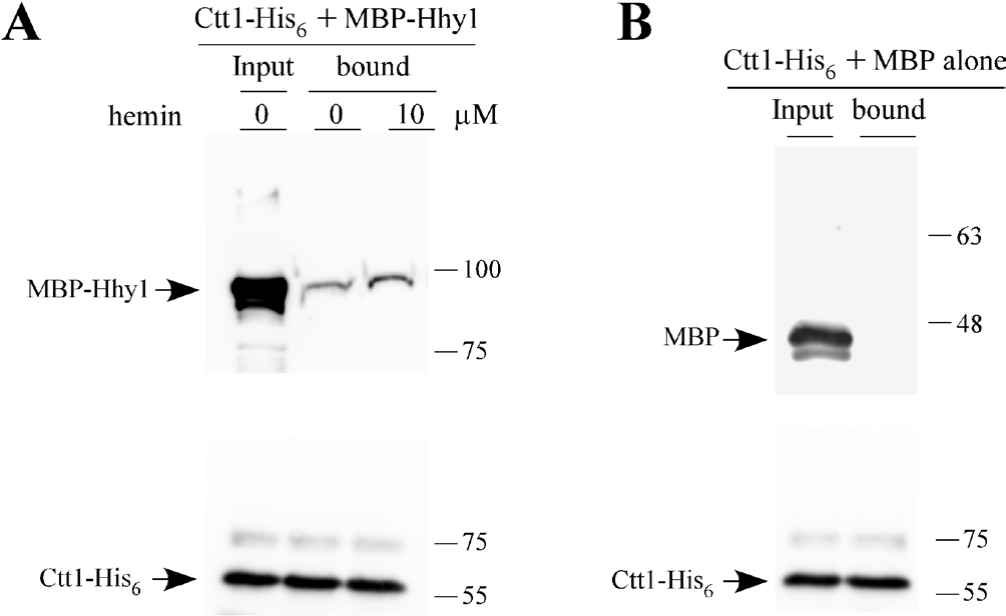
Bacterially expressed Ctt1‐His_6_ and MBP‐Hhy1 directly interact with one another in vitro. (A, B) Pull‐down interaction assays were performed using purified Ctt1‐His_6_ and either MBP‐Hhy1 or MBP alone. Purified MBP‐Hhy1 or MBP was incubated with Ni^2+^‐nitrilotriacetic acid agarose beads in the presence of Ctt1‐His_6_, either without hemin supplementation or supplemented with hemin (10 μM). After washes, the bound fractions (IP) were eluted with imidazole (150 mM) and analyzed by immunoblot assays using anti‐His_6_ and anti‐MBP antibodies. Aliquots of each purified protein (Input) were analyzed prior to chromatography to confirm protein integrity. Molecular weight markers (kDa) are indicated on the right side of each panel for reference.

Given the interaction detected between Hhy1 and Ctt1 in coimmunoprecipitation experiments using yeast cell extracts, we further investigated whether purified, bacterially expressed forms of these proteins could directly interact. Either MBP alone or the MBP‐Hhy1 fusion protein was incubated with Ctt1‐His_6_, which had been immobilized on Ni^2+^‐nitrilotriacetic acid‐agarose beads. The protein mixtures were incubated with the beads and rotated end‐over‐end for 2 h, either in the absence or presence of hemin (10 μM). After washes, bound His_6_‐tagged Ctt1 and any associated proteins were eluted with imidazole and analyzed by immunoblotting using anti‐His_6_ and anti‐MBP antibodies. Results showed that both Ctt1‐His_6_ and MBP‐Hhy1 were present in the immunoprecipitated (bound) fraction (Figure 9A). Notably, the detection of MBP‐Hhy1 exhibited a slight enrichment when the incubation was performed in the presence of hemin compared to the condition without hemin supplementation (Figure 9A). To validate the specificity of the coimmunoprecipitation, MBP alone was not retained by Ctt1‐His_6_ and was absent from the bound fractions (Figure 9B). Together, these results indicated that Ctt1 and Hhy1 directly interact with one another and that no other protein is required to mediate this interaction.

## 3 Discussion

In this study, we generated an *S. pombe hem1*Δ *sib1*Δ *sib2*Δ *fio1*Δ *fip1*Δ mutant strain that is unable to acquire inorganic iron through its high‐affinity reductive iron uptake system and cannot synthesize and secrete Fc. The goal was to minimize potential interference from these pathways with cellular acquisition and scavenging of exogenous heme. The presence of the *hhy1* ^+^ gene immediately adjacent to the *str3* ^+^ and *shu1* ^+^ genes on chromosome I, together with its iron‐repressed expression in response to elevated iron concentrations, suggested a role for the Hhy1 protein in iron or heme homeostasis.

In *Candida albicans* , no Hhy1‐like protein has been identified; however, putative Hhy1 orthologs are present in other members of the *Saccharomycetales* order, such as *Metschnikowia bicuspidata, Clavispora lusitaniae*, and *Komagataella pastoris* (Rutherford et al. 2024). Although an Hhy1 ortholog also appears to be absent in *Cryptococcus neoformans* , a putative Hhy1 ortholog has been identified in *Histoplasma capsulatum*, which belongs to the same *Onygenales* order as *Paracoccidioides brasiliensis* and *P. lutzii* . Both *H. capsulatum* and *Paracoccidioides* species are able to use exogenous heme as an iron source (Kornitzer and Roy 2020).

Using cell proliferation assays, we found that deletion of *hhy1* ^+^ (*hhy1*Δ) causes a heme‐dependent growth defect in *hem1*Δ *sib1*Δ *sib2*Δ *fio1*Δ *fip1*Δ cells. In our previous studies using the *hem1*Δ‐based approach, the fluorescent heme analog ZnMP proved to be a highly practical tool for assessing the ability of *S. pombe* strains to store and distribute hemin over time in living cells (Mourer et al. 2015, 2017; Normant et al. 2018). Accordingly, to further investigate the effect of *hhy1* ^+^ inactivation in *hem1*Δ *sib1*Δ *sib2*Δ *fio1*Δ *fip1*Δ cells, these mutant cells were incubated in ALA‐free medium supplemented with ZnMP under low‐iron conditions. Unexpectedly, the addition of fluorescent ZnMP to *hem1*Δ *sib1*Δ *sib2*Δ *fio1*Δ *fip1*Δ *hhy1*Δ cells produced a sustained ZnMP fluorescence signal compared to Hhy1‐expressing *hem1*Δ *sib1*Δ *sib2*Δ *fio1*Δ *fip1*Δ cells. Consistent with this observation, *hem1*Δ *sib1*Δ *sib2*Δ *fio1*Δ *fip1*Δ *hhy1*Δ cells exhibited 40%–50% higher intracellular heme levels than the corresponding Hhy1‐expressing strain, indicating that elevated intracellular heme is not necessarily beneficial nor readily available in a usable form to support hemin‐dependent growth. At present, the molecular basis underlying heme persistence in *hem1*Δ *sib1*Δ *sib2*Δ *fio1*Δ *fip1*Δ cells lacking Hhy1 remains unclear. One possibility is that the concentration of labile heme is increased due to a reduced fraction of inert heme being sequestered within intracellular compartments or bound to abundant hemoproteins. A second possibility is that *hem1*Δ *sib1*Δ *sib2*Δ *fio1*Δ *fip1*Δ *hhy1*Δ cells retain ZnMP more efficiently, suggesting a potential role for Hhy1 in ZnMP catabolism or export. At the zero time point, no difference in ZnMP uptake was observed between *hhy1* ^+^‐expressing cells and *hhy1Δ* cells. These results may be explained by the possibility that, despite performing an extended wash after ZnMP or hemin incubation, residual ZnMP or hemin remained nonspecifically associated with the cell surface, thereby obscuring potential differences in uptake between *hhy1* ^+^ and *hhy1Δ* cells. Nonetheless, taken together, these findings suggest that Hhy1 may function to limit the cytotoxic effects of excess labile heme when its cellular concentration becomes abnormally high.

These results are reminiscent of the increased labile heme pool observed in *S. cerevisiae* cells lacking the glyceraldehyde‐3‐phosphate dehydrogenase (GAPDH) Tdh3, compared with wild‐type cells expressing Tdh3 (Hanna et al. 2016). Deletion of Tdh3 results in a fourfold increase in labile heme levels, as monitored using the ratiometric fluorescent heme sensor HS1‐M7A (Hanna et al. 2016). In *S. cerevisiae* , Tdh3 is known to buffer cytosolic heme and may serve as an intracellular heme reservoir that regulates the activity of the nuclear heme‐dependent transcription factor Hap1 (Hanna et al. 2016). However, the mechanism by which cytosolic Tdh3 shuttles between the cytosol and nucleus to deliver and transfer heme to Hap1 remains unclear. Although the concentration of labile cytosolic heme is higher in *S. cerevisiae tdh3*Δ mutant cells, these cells maintain normal activity of the cytosolic heme catalase Ctt1, in contrast to the dysregulation of the transcription factor Hap1 (Hanna et al. 2016). This role of *S. cerevisiae* Tdh3 differs from that of Hhy1, whose disruption decreases Ctt1 activity by 39% under hemin‐dependent growth conditions.

Based on our results, Hhy1 does not appear to promote Ctt1 activity when cells were grown in ALA‐free medium and endogenous heme biosynthesis was subsequently restored by ALA addition. One potential explanation for the difference in the bioavailability of endogenous (+ALA) versus exogenous (−ALA) heme in promoting Ctt1 activity is that heme trafficking could differ depending on whether heme is synthesized intracellularly or imported from the environment. In *S. pombe*, at least two independent routes for heme acquisition (the Shu1‐ and Str3‐dependent pathways) operate in addition to de novo heme synthesis. At present, it remains unknown whether Hhy1 is a hemoprotein target supplied by the Shu1‐ or Str3‐mediated heme acquisition pathway. However, based on our results, Hhy1 does not appear to be efficiently supplied by endogenously synthesized heme when biosynthesis was subsequently restored by ALA addition. These findings in *S. pombe* are reminiscent of those reported in *Mycobacterium smegmatis* and *M. tuberculosis* , in which distinct mechanisms allow differential handling of exogenous and endogenous heme, as well as differential intracellular heme trafficking for hemoprotein activation (Donegan et al. 2022).

Studies of human GAPDH, the human orthologue of Tdh3, have shown that the His^53^ residue is required for the heme binding property of GAPDH (Sweeny et al. 2018). This residue corresponds to His^51^ in *S. cerevisiae* Tdh3. Consistent with this, GAPDH H53A and Tdh3 H51A variants lose their ability to bind heme (Sweeny et al. 2018). When these variants are expressed in *S. cerevisiae tdh3*Δ cells, they fail to rescue the *tdh3*Δ phenotype, which is characterized by a fourfold increase in labile heme levels (Sweeny et al. 2018). Furthermore, expression of the GAPDH H53A and Tdh3 H51A variants in *tdh3*Δ cells results in markedly reduced heme‐dependent Hap1 activity (Sweeny et al. 2018). However, how coordinated heme involving the specific His53 or His51 residue from GAPDH or Tdh3 is transferred to downstream client hemoproteins, including Hap1, remains unknown.

In the case of Hhy1, two truncated forms, Hhy1MutA(1–261) and Hhy1MutB(262–463), were expressed and purified from *E. coli* . Although the Hhy1MutB variant displayed a 17.1‐fold lower affinity for hemin compared to wild‐type Hhy1, it nevertheless retained hemin‐binding capacity, whereas this property was lost in the Hhy1MutA mutant. Within the 262–463 amino acid region of Hhy1, we identified a CPC motif (Cys^321^‐Pro^322^‐Cys^323^) required for maximal hemin binding by Hhy1. This differs from GAPDH or Tdh3, in which an His residue, rather than a CP motif, serves as the hemin‐binding site (Sweeny et al. 2018). In several prokaryotic and eukaryotic hemoproteins, the CP motif functions as the hemin‐binding determinant (Shimizu et al. 2019). Notably, the Pro residue adjacent to Cys promotes an open‐access conformation of the Cys thiol group, thereby facilitating heme coordination when this thiol becomes more exposed within the predicted 3D protein structure.

Consistent with this notion, mutation of the Cys^321^, Pro^322^, and Cys^323^ residues within full‐length Hhy1, which disrupts the CPC motif (including the Cys^321^‐Pro^322^ motif), reduced hemin binding by 7.4‐fold relative to the wild‐type protein. However, this was not a complete loss of hemin‐binding activity, meaning that additional amino acids contribute to hemin binding by Hhy1. In contrast, mutation of the Cys^290^‐Pro^291^ motif did not significantly affect hemin binding, with a *K*_*D*_ value of 1.44 μM compared to 1.09 μM for the wild‐type protein. Therefore, identification of additional residues required for maximal hemin binding to Hhy1 will necessitate further site‐directed mutagenesis studies.

*S. pombe* possesses a single monofunctional heme‐containing catalase, named Ctt1. We found that Ctt1 displays a pancellular localization, consistent with what has been reported previously (Matsuyama et al. 2006). This situation differs somewhat from that in *S. cerevisiae* , which contains two monofunctional heme‐containing catalases, Cta1 and Ctt1 (Engel et al. 2025). In *S. cerevisiae* , Cta1 localizes to the peroxisomal and mitochondrial matrices (Petrova et al. 2004), whereas Ctt1 localizes in the cytoplasm (Koh et al. 2015). Both enzymes are functionally similar and catalyze the degradation of H_2_O_2_. The amino acid sequence of *S. pombe* Ctt1 shares 44.3% and 52.3% identity with *S. cerevisiae* Ctt1 and Cta1, respectively, consistent with its reported catalase activity (Mutoh et al. 1999). Given that *S. pombe* Ctt1 is present in the cytoplasm, similar to *S. cerevisiae* Ctt1, we next assessed whether its activity was altered in the absence of the cytoplasmic hemoprotein Hhy1. The *hem1*Δ *sib1*Δ *sib2*Δ *fio1*Δ *fip1*Δ *hhy1*Δ cells exhibited a 39% reduction in Ctt1 activity when grown in ALA‐free medium supplemented with Dip and hemin, as measured by a spectrophotometric liquid catalase assay. Similarly, *S. pombe* catalase activity is reduced (47%) in extracts from *hem1Δ hhy1Δ* cells compared with *hem1Δ* cells expressing the endogenous *hhy1* ^+^ gene (Figure S1). Further analyses using bimolecular fluorescence complementation (BiFC) and coimmunoprecipitation assays showed that Hhy1 and Ctt1 physically interact, both in *S. pombe* cells and when using bacterially expressed proteins. However, given that typical catalases are structurally complex homo‐tetrameric enzymes with a single heme buried within each subunit (Chelikani et al. 2004), it remains unclear whether Hhy1 adopts a dimeric or higher‐order multimeric form to interact with Ctt1 in yeast cells. Moreover, since our data indicate that Hhy1 buffers cytosolic heme and influences Ctt1 activity, it is possible that Hhy1 serves as a source of heme. Nevertheless, Ctt1 does not rely exclusively on Hhy1, as its activity is still detectable in cells lacking Hhy1.

In vitro studies investigating the assembly of the *Enterococcus faecalis* catalase KatA from its apoform have shown that the initial step involves hemin binding to a partially folded monomeric form of KatA (Baureder et al. 2014). This is followed by oligomerization of hemylated KatA monomers into an enzymatically active tetramer (Baureder et al. 2014). By analogy with *S. pombe* Ctt1, an alternative role for Hhy1 may be to facilitate the assembly of an active Ctt1 homo‐tetrameric complex, thereby promoting full catalase activity.

The apocatalase KatA exhibits a high affinity for hemin with a *K*_*D*_ of 0.15 μM, which is considerably stronger than the hemin affinities reported for other classes of hemoproteins, such as Shu1, Str3, Hap1, and Gis1 (Mourer et al. 2015; Normant et al. 2018; Shimizu et al. 2019). Assuming that the hemin *K*_*D*_ value for *S. pombe* Ctt1 is similar to that of KatA, this would suggest that Ctt1 has a higher hemin‐binding affinity than Hhy1 (*K*_*D*_ of 1.09 μM). This is consistent with Ctt1 acting downstream in hemin capture after its transport into the cell, where heme would first transiently associate with lower‐affinity proteins such as Hhy1. In this model, Hhy1 would function as a cytosolic heme‐buffering or reservoir factor for inert heme that is ultimately delivered to higher‐affinity hemoproteins such as catalases and cytochromes.

There are at least two advantages conferred by Hhy1 in *hem1*Δ *sib1*Δ *sib2*Δ *fio1*Δ *fip1*Δ cells grown under hemin‐dependent conditions. First, these cells exhibit robust growth when hemin is provided as the sole source of heme under low‐iron conditions. Second, they show increased resistance to H_2_O_2_‐induced oxidative stress, which is consistent with the requirement for Hhy1 in supporting full heme‐dependent activation of Ctt1. Further studies will be needed to elucidate the mechanisms by which Hhy1 acquires heme and contributes to the heme‐dependent activation of the catalase Ctt1.

## 4 Experimental Procedures

### 4.1 Yeast Strains, Media, and Growth Conditions

The genotypes of the *S. pombe* strains used in this study are listed in Table 1. All strains were derived from the reference strain FY435, in which the *hem1* ^+^ gene was disrupted (TMY1), generating a series of ALA‐auxotrophic strains. Under standard growth conditions, *hem1*Δ‐related strains were cultured in yeast extract plus supplements (YES) medium (Sabatinos and Forsburg 2010) supplemented with the indicated concentrations of ALA. For growth in the absence of ALA, YES medium was supplemented with exogenous hemin, forcing the strains to rely on exogenous heme uptake for growth. For plasmid transformation, cells were grown in Edinburgh minimal medium (EMM) supplemented with ALA and lacking the required amino acids for plasmid selection.

**TABLE 1:** *S. pombe* strains used in this study.

Strain	Genotype	Source or references
FY435	*h* ^+^ *his7‐366 leu1‐32 ura4‐*Δ*18 ade6‐M210*	Mourer et al. (2015)
TMY1	*h* ^+^ *his7‐366 leu1‐32 ura4‐*Δ*18 ade6‐M210 hem1*Δ::*KAN* ^*r*^	Mourer et al. (2015)
TVY1	*h* ^+^ *his7‐366 leu1‐32 ura4‐D18 ade6‐M210 hem1*Δ::*loxP fep1*Δ::*KAN* ^*r*^	Vahsen et al. (2023)
TVY8	*h + his7‐366 leu1‐32 ura4‐D18 ade6‐M210 hem1*Δ::*loxP php4*Δ::*loxP fep1*Δ::*KAN^r^*	Vahsen et al. (2023)
TVY9	*h* ^+^ *his7‐366 leu1‐32 ura4‐*Δ*18 ade6‐M210 hem1*Δ::*loxP sib1sib2*Δ::*loxP fio1fip1*Δ::*KAN* ^*r*^	This study
TVY10	*h* ^+^ *his7‐366 leu1‐32 ura4‐*Δ*18 ade6‐M210 hem1*Δ::*loxP sib1sib2*Δ::*loxP fio1fip1*Δ::*loxP fep1*Δ::*KAN* ^*r*^	This study
TVY11	*h* ^+^ *his7‐366 leu1‐32 ura4‐*Δ*18 ade6‐M210 hem1*Δ::*loxP sib1sib2*Δ::*loxP fio1fip1*Δ::*loxP hhy1*Δ::*KAN* ^*r*^	This study
TVY12	*h* ^+^ *his7‐366 leu1‐32 ura4‐*Δ*18 ade6‐M210 hem1*Δ::*loxP sib1sib2*Δ:*:loxP fio1fip1*Δ::*loxP hhy1*Δ::*KAN* ^*r*^ *ctt1‐mCherry*::*HPH* ^*r*^	This study
TVY13	*h* ^+^ *his7‐366 leu1‐32 ura4‐*Δ*18 ade6‐M210 hem1*Δ::*loxP sib1sib2*Δ::*loxP fio1fip1*Δ::*KAN* ^*r*^ *ctt1‐mCherry*::*HPH* ^*r*^	This study
TVY14	*h* ^+^ *his7‐366 leu1‐32 ura4‐*Δ*18 ade6‐M210 hem1*Δ::*loxP sib1sib2*Δ::*loxP fio1fip1*Δ::*loxP hhy1*Δ::*KAN* ^*r*^ *ctt1‐VC*::*HPH* ^*r*^	This study
TVY15	*h* ^+^ *his7‐366 leu1‐32 ura4‐*Δ*18 ade6‐M210 hem1*Δ::*loxP sib1sib2*Δ::*loxP fio1fip1*Δ::*loxP ctt1*Δ::*KAN* ^*r*^	This study

For cell proliferation assays, precultures of the specified strains were grown in YES medium supplemented with ALA (200 μM) and FeCl_3_ (50 μM). Upon reaching mid‐logarithmic phase, cells were treated with Dip (250 μM) for 3 h. Cells were then washed twice and diluted 1000‐fold into ALA‐free medium containing 75 μM Dip. The diluted cultures were divided into three treatment groups: (i) ALA (200 μM) and FeCl_3_ (100 μM); (ii) ALA (200 μM) and Dip (75 μM); and (iii) hemin (0.25 μM) and Dip (75 μM). At this stage (zero time point), cell growth was monitored by measuring optical density at 600 nm (OD_600_) at the indicated times.

For assessing cell growth sensitivity to H_2_O_2_, precultures of the indicated strains were cultured to mid‐logarithmic phase in YES medium supplemented with ALA (25 μM) and FeCl_3_ (25 μM). Cultures were then washed twice and divided into three experimental conditions. One condition contained 100 μM ALA and 25 μM Dip, whereas the other two lacked ALA but contained 25 μM Dip. After 4 h 30 min of incubation, hemin (1 μM) was added to one of the ALA‐free groups, and all three groups were further incubated for 90 min. Cells from each condition were then spotted in serial dilutions (500 cells/10 μL and 50 cells/10 μL) onto YES plates containing either 200 μM ALA alone or ALA plus 0.2 mM H_2_O_2_. Plates were incubated for 3 days at 30°C and photographed.

### 4.2 Plasmids

The *hhy1* ^+^ gene was amplified by PCR using primers designed to encompass the *hhy1* ^+^ locus from −1497 bp upstream of the initiator codon up to the stop codon. The resulting PCR product was digested with ApaI and NotI and inserted into the corresponding sites of pJK148 (Keeney and Boeke 1994), generating plasmid pJK‐1497*hhy1* ^+^. An 18‐bp StuI‐BspEI linker was inserted in‐frame within the *hhy1* ^+^ coding sequence at position +147 relative to the first nucleotide of the initiator codon. The linker was introduced using an overlap extension PCR method (Ho et al. 1989). This insertion resulted in the addition of six extra amino acid residues following the Lys residue at position 49, located within a predicted hydrophilic region of Hhy1. The resulting DNA construct was digested with StuI and BspEI to replace the linker region with the coding sequence of GFP, mNeonGreen, or the Venus amino‐terminal fragment (VN), each of which had been amplified by PCR using primers containing StuI and BspEI sites. The resulting plasmids were named pJK‐1497*hhy1* ^+^‐GFP, pJK‐1497*hhy1* ^+^‐mNG, and pJK‐1497*hhy1* ^+^‐VN, respectively.

To generate strains in which the mCherry or Venus carboxyl‐terminal fragment (VC) coding sequence was inserted downstream of and in‐frame with the 3′ chromosomal region of *ctt1* ^+^, a PCR‐based gene fusion strategy was used. This approach was adapted from the method employing the pFA6A‐mCherry‐kanMX6 module (Malcova et al. 2016), except that the hygromycin (HPH) selection marker was used instead of kanMX6. This strategy enabled site‐specific integration of mCherry or VC at the chromosomal *ctt1* ^+^ locus, resulting in the replacement of the wild‐type allele with an mCherry‐ or VC‐tagged *ctt1* ^+^ allele.

The coding sequence of the maltose‐binding protein (MBP) was fused to the N terminus of Hhy1 or its mutant derivatives, such as MutA(1–261) and MutB(262–463). To generate these fusions, each DNA fragment was amplified by PCR using pJK‐1497*hhy1* ^+^ as the template. The purified fragments were digested with BamHI and SalI and then cloned into the corresponding sites of pMAL‐c2x, producing pMAL‐Hhy1, pMAL‐Hhy1MutA, and pMAL‐Hhy1MutB. Site‐specific Hhy1 mutants (C290A, P291A; C321A, P322A, C323A; and C290A, P291A + C321A, P322A, C323A) were generated by overlap extension PCR using pMAL‐Hhy1 as the template. The *ctt1* ^+^ coding sequence was amplified with primers introducing NcoI and NotI restriction sites at the termini of the PCR product. The purified fragment was inserted into the corresponding sites of pET28a, generating pET28a‐*ctt1* ^+^ *‐His*_*6*_. Plasmids pJK‐1478*fep1* ^+^, pJK‐1478*NTAP‐fep1* ^+^, and pBP*ctr4* ^+^ *‐VC* have been described previously (Ioannoni et al. 2010; Pelletier et al. 2002, 2005).

### 4.3 RNA Isolation and Quantitative Real‐Time Reverse Transcription PCR Analysis

Total RNA was extracted from cell cultures using a hot phenol method, as previously described (Chen et al. 2003). Reverse transcription, cDNA synthesis, and quantitative PCR (qPCR) were performed following procedures, as previously described (Brault et al. 2022). Each target transcript (*hhy1* ^+^, *srx1* ^+^, or *act1* ^+^) was analyzed in experiments conducted with three independent biological replicates, with each sample reaction performed in triplicate. Results were considered valid when the target‐specific fluorescent signal exhibited a *C*_t_ value ≤ 37 cycles, and when positive and negative control reactions showed productive amplification and no amplification, respectively. Fold changes in transcript levels in strains expressing Fep1 or lacking Fep1 (*fep1*Δ) were calculated using the ΔΔCt method, with normalization to the internal reference transcript *act1* ^+^ (Livak and Schmittgen 2001; Protacio et al. 2022; Schmittgen and Livak 2008). The following equation was applied: ΔΔCt = [(Ct gene‐Ct ref) in *hem1*Δ *fep1* ^+^] versus [(Ct gene‐Ct ref) in *hem1*Δ *fep1*Δ]; or, ΔΔCt = [(Ct gene‐Ct ref) in *hem1*Δ *sib1*Δ *sib2*Δ *fio1*Δ *fip1*Δ *fep1* ^+^] versus [(Ct gene‐Ct ref) in *hem1*Δ *sib1*Δ *sib2*Δ *fio1*Δ *fip1*Δ *fep1*Δ], under the indicated experimental conditions. The positions of the amplified regions for each transcript relative to the first nucleotide of the initiation codon were as follows: *hhy1* ^+^, +493 to +596; *srx1* ^+^, +178 to +274; and, *act1* ^+^, +173 to +280.

### 4.4 ChIP Assays

Precultures of *hem1*Δ *php4*Δ *fep1*Δ cells expressing either an untagged or a TAP‐tagged *fep1* ^+^ allele were grown in the presence of ALA (200 μM) and FeCl_3_ (50 μM). When cultures reached an OD_600_ of 0.5, cells were washed and transferred to an ALA‐free medium containing FeCl_3_ (50 μM) for 5 h. ALA‐starved cells were then washed and incubated in YES medium supplemented with hemin (5 μM) and treated with either Dip (300 μM) or FeCl_3_ (Fe, 100 μM) for 3 h. Cells were subsequently fixed with formaldehyde, neutralized with glycine, and lysed by glass bead disruption, as previously described (Brault et al. 2016; Larochelle et al. 2012). The resulting lysates were sonicated to shear chromatin DNA into fragments of approximately 600–1000 bp. Immunoprecipitation of TAP‐tagged Fep1 bound to chromatin was performed using immunoglobulin G (IgG)‐Sepharose beads, following the procedures as previously described (Jacques et al. 2014). Bead handling, including washing, elution, cross‐link reversal, and DNA precipitation, was carried out as previously described (Adam et al. 2001; Jbel et al. 2009). Quantification of immunoprecipitated DNA was conducted by quantitative real‐time PCR (qPCR) using primer sets targeting the promoter regions of *hhy1* ^+^ and *srx1* ^+^. The occupancy of TAP‐Fep1 at these loci was determined by calculating the enrichment of the *hhy1* ^+^ and *srx1* ^+^ promoter regions relative to a GATA‐free 18S ribosomal DNA coding region, which served as an internal background control. The primer pairs used were as follows: *hhy1* ^+^‐1331 (5′‐TTCGATTATTGGGTTGGCTTT‐3′)/*hhy1* ^+^‐1211 (5′‐CTTTGCTAAACGTAAACACAAGG‐3′), and *srx1* ^+^‐209 (5′‐ATATACATACCGGGATAAGATAGGTTG‐3′)/*srx1* ^+^‐134 (5′‐TACTCCTTGATCTGATATCGTTTCC‐3′). Primers used for amplifying the 18S ribosomal DNA coding region were described previously (Brault et al. 2016). Each qPCR reaction was performed in triplicate using the Perfecta SYBR Green Fast mix (Quanta) on a CFX96 Touch Real‐Time PCR instrument (BioRad). All ChIP experiments were independently repeated three times with separate chromatin preparations.

### 4.5 Protein Extraction and Coimmunoprecipitation Assays

To analyze the steady‐state levels of Hhy1‐GFP expressed in *hem1∆ sib1∆ sib2∆ fio1∆ fip1∆ hhy1∆* and *hem1∆ sib1∆ sib2∆ fio1∆ fip1∆ hhy1∆ fep1∆* strains, whole cell extracts were prepared using glass beads and a FastPrep‐24 instrument (MP Biomedicals, Solon, OH). Cells were lysed in a modified TMN_150_ buffer containing 50 mM Tris–HCl (pH 7.5), 150 mM NaCl, 5 mM MgCl_2_, 0.1% Nonidet P‐40, 0.2% sodium dodecyl sulfate (SDS), 1 mM phenylmethylsulfonyl fluoride (PMSF), and a complete protease inhibitor cocktail (P8340, Sigma‐Aldrich). In the case of *hem1∆ sib1∆ sib2∆ fio1∆ fip1∆* cells expressing Ctt1‐mCherry and *hem1∆ sib1∆ sib2∆ fio1∆ fip1∆ hhy1∆* cells co‐expressing Ctt1‐mCherry and Hhy1‐mNeonGreen, PMSF (1 mM) was added directly to the cultures 10 min before harvesting. Extracts were prepared in HEGN_100_ lysis buffer containing 20 mM 4‐(2‐hydroxyethyl)‐1‐piperazineethanesulfonic acid (HEPES), pH 7.9, 100 mM NaCl, 1 mM ethylenediaminetetraacetic acid (EDTA), 10% glycerol, 0.1 mM Na_3_VO_4_, 1 mM PMSF, 1 mM dithiothreitol (DTT), and a complete protease inhibitor mixture. Equal amounts of protein extracts were resolved on 8% SDS‐polyacrylamide gels and analyzed by immunoblot assays as described previously (Brault et al. 2016). For the detection of Hhy1‐GFP, Ctt1‐mCherry, and α‐tubulin, the following primary antibodies were used: monoclonal anti‐GFP antibody B‐2 (Santa Cruz Biotechnology), polyclonal anti‐mCherry antibody E5D8F (Cell Signaling Technology), and monoclonal anti‐α‐tubulin antibody B‐5‐1‐2 (Sigma‐Aldrich). After incubation with the primary antibodies, membranes were washed and incubated with the appropriate horseradish peroxidase (HRP)‐conjugated secondary antibodies (Amersham Biosciences). Protein signals were detected using enhanced chemiluminescence (ECL) reagents (Amersham Biosciences) and visualized with an ImageQuant LAS 4000 instrument (GE Healthcare). For quantitative western blot analyses, membranes were probed with an IRDye 800CW goat anti‐rabbit‐IgG secondary antibody and a DyLight 680‐conjugated goat anti‐mouse secondary antibody, followed by detection using an Odyssey infrared imaging system (LI‐COR).

Coimmunoprecipitation assays were performed as previously described (Ping et al. 2024), except that whole‐cell extracts were incubated for 4 h with monoclonal anti‐mCherry antibodies bound to protein G‐Sepharose 4 Fast Flow beads (Sigma, GE17‐0618‐01). Following end‐over‐end mixing on a rotating wheel at 4°C, the beads were washed, and the bound proteins were eluted as previously described (Ping et al. 2024). The resulting immunoprecipitated proteins were then analyzed by immunoblotting, as described above for the western blot analysis of proteins detected from whole‐cell extract preparations.

### 4.6 Catalase Activity Assays

Whole‐cell protein extraction was performed with glass beads in HEGN_100_ lysis buffer. For in‐gel activity assays, equal amounts of protein extracts were resolved on 4% native polyacrylamide gels at 4°C. After electrophoresis, the native gels were washed twice for 5 min in ultrapure water, incubated for 10 min in a freshly prepared H_2_O_2_ solution (4 mM), and then washed twice again in ultrapure water. Subsequently, the gels were incubated with gentle shaking for 1 min in a freshly prepared solution containing ferric chloride (61.7 mM) and potassium ferricyanide (27.2 mM) (Lorusso et al. 2022; Pezzoni et al. 2018). In‐gel catalase activity was visualized as achromatic bands against a bluish background, resulting from the disproportionation of H_2_O_2_ and the dissolution of Prussian blue within the gel. After washing the gels with 10 volumes of water, they were visualized using a documentation imaging system.

Spectrophotometric determination of catalase activity was also carried out using equal amounts of protein extracts mixed with P buffer (100 mM sodium phosphate buffer, pH 8.0, containing 0.001% Triton X‐100) and incubated in the presence of H_2_O_2_ (16.6 mM). The disproportionation of H_2_O_2_ was measured by monitoring the decrease in absorbance at 240 nm over a 3‐min period. In parallel, an H_2_O_2_ standard curve was established under identical conditions using known H_2_O_2_ concentrations and their corresponding spectrophotometric readings. This calibration enabled quantification of the percentage of H_2_O_2_ disproportionation attributable to catalase activity in the analyzed samples.

### 4.7 Fluorescence Microscopy

Fluorescence and differential interference contrast (Nomarski) images of cells were acquired using a Nikon Eclipse E800 epifluorescence microscope (Nikon, Melville, NY) equipped with a Hamamatsu ORCA‐ER cooled digital camera (Hamamatsu, Bridgewater, NJ). Cells were visualized at 1000× magnification using two filter sets: 465–495 nm (for GFP and mNeonGreen signals) and 510–560 nm (for Cherry and ZnMP signals). Representative cell fields presented in this study were obtained from at least five independent experiments. Furthermore, the displayed images reflect protein localization patterns observed in 200 cells analyzed per condition.

### 4.8 ZnMP Detection

The indicated strains were precultured in the presence of ALA, washed and then transferred to an ALA‐free medium containing Dip (25 μM) and hemin (1 μM) for 1.5 h. Cultures were then divided into two groups, either left untreated or supplemented with ZnMP (10 μM) for an additional 1.5 h. After this incubation, cells were harvested and washed twice with phosphate‐buffered saline (PBS) containing bovine serum albumin (BSA; 5 mg/mL). The washed cells were transferred to fresh ALA‐free medium containing Dip (25 μM). At the zero time point and after 1.5, 3, and 19 h, aliquots were analyzed by fluorescence microscopy to detect ZnMP fluorescence. Fluorescence intensity was quantified using ImageJ software (v1.53e).

### 4.9 Total Heme Measurements

The indicated strains were precultured in the presence of ALA, washed and then transferred to an ALA‐free medium containing Dip (25 μM) for 1.5 h. Cultures were then divided into two groups, either left untreated or supplemented with hemin (0.25 μM). At this point (zero time) and after 3 and 19 h, aliquots were washed and then resuspended in oxalic acid (20 mM), and kept at 4°C in the dark for 16 h, as previously described (Hanna et al. 2018; Michener et al. 2012). After the incubation period, an equal volume (500 μL) of oxalic acid (2 M) was added to each sample. The sample suspensions were then split, with one half incubated at 98°C for 30 min, whereas the other half was maintained at 25°C for the same duration. All samples were centrifuged for 2 min at maximum speed in a tabletop microcentrifuge, and porphyrin fluorescence (excitation: 400 nm; emission: 620 nm) was measured using a fluorometric assay, as previously described (Hanna et al. 2018; Michener et al. 2012). Additionally, heme concentrations were calculated from a standard curve generated using hemin chloride stock solutions, following the methodology described in previous studies (Hanna et al. 2018; Hans et al. 2001; Michener et al. 2012; Sassa 1976).

### 4.10 Absorbance Spectroscopy

A stock solution of hemin (25 mM) was freshly prepared in 0.1 M sodium hydroxide (NaOH). The solution was filtered and then diluted 1:1000. The concentration of hemin was determined by measuring absorbance at 385 nm using an extinction coefficient of 58,400 L·mol^−1^·cm^−1^. To assess the interaction between hemin and wild‐type Hhy1 or its mutant derivatives, increasing concentrations of purified protein (0–5 or 0–10 μM) were added to hemin (10 μM) in buffer H (20 mM HEPES, pH 7.4, and 40% dimethyl sulfoxide). Absorbance spectra were recorded from 350 to 700 nm. Formation of the protein‐hemin complex was monitored by decreases in absorbance at the Soret peak. Graphs were generated to illustrate changes in absorbance at the Soret peak as a function of protein concentration. Changes in absorbance (ΔA) at 402 nm measured for wild‐type and mutant forms of Hhy1 were plotted as a function of indicated protein concentrations, and *K*_*D*_ values were determined using a ‘one site binding’ model in Prism software (GraphPad, v10.6.1) by least‐squares fitting of a saturation binding curve to the data.

### 4.11 Expression and Purification of MBP‐Hhy1, Its Mutant Derivatives, and Ctt1‐His_6_ in *E. coli*

The pMAL expression plasmids containing the coding sequences of Hhy1 and its mutant derivatives fused to MBP were transformed into *E. coli* Rosetta(DE3)pLysS cells. Cultures were grown to an OD_600_ of 0.5, and protein expression was induced with isopropyl‐β‐D‐thiogalactopyranoside (IPTG, 0.2 mM) for 20 h at 22°C in the presence of ethanol (2%). Cells were harvested and sonicated in buffer A (50 mM Tris–HCl, pH 7.5, 150 mM NaCl, 10% sucrose, 50 μg/mL lysozyme, and 1% Triton X‐100) supplemented with protease inhibitors. The lysates were incubated with amylose resin for 16 h at 4°C and washed with buffer A. MBP‐Hhy1 and its mutant derivatives bound to the resin were eluted using buffer A containing 25 mM maltose. Eluted fractions containing enriched purified proteins were dialyzed to remove maltose and, when necessary, subjected to an additional round of purification using the same affinity resin.

For expression of Ctt1‐His_6_, the *ctt1* ^+^ coding sequence was cloned into pET28a and transformed into the same *E. coli* strain. Growth conditions were similar to those used for Hhy1 expression, except that induction in the presence of IPTG (0.2 mM), ethanol (2%), and hemin (10 μM) was performed at 37°C for 90 min. Cells were disrupted by sonication in buffer A, and the resulting lysates were incubated with nickel‐nitrilotriacetic acid‐agarose (Ni‐NTA) beads for 19 h at 4°C. Beads were then washed with buffer A containing 50 mM imidazole, and bound proteins were eluted stepwise with buffer B (50 mM Tris–HCl, pH 7.5, 300 mM NaCl, 10% glycerol) containing increasing concentrations of imidazole, as described previously (Normant et al. 2018). Fractions eluted with 1000 mM imidazole were dialyzed to remove imidazole and subjected to an additional round of purification using the same type of affinity resin.

To assess the ability of purified, bacterially expressed Ctt1‐His_6_ to interact with either MBP‐Hhy1 or MBP alone, the proteins were mixed in phosphate‐buffered saline containing Tween 20 (0.1%), imidazole (20 mM) (PBSTI), with or without 10 μM hemin as indicated. After incubation for 1 h at 4°C, Ni‐NTA agarose beads were added, and the mixtures were rotated end‐over‐end for an additional 2 h at 4°C. The beads were then washed three times with 1.5 mL of PBSTI, transferring them to a fresh microtube before the final wash. The immunoprecipitates were resuspended in SDS loading buffer (240 mM Tris–HCl, pH 6.8, 40% glycerol, 8% SDS, 2.8 mM β‐mercaptoethanol, 1 mM DTT, 6 M urea, and 2 M thiourea) supplemented with 200 mM imidazole and heated at 65°C for 20 min. Samples were resolved on 8% SDS‐polyacrylamide gels and analyzed by western blotting using monoclonal anti‐MBP antibody E8032L (New England Biolabs) and monoclonal anti‐His antibody 34460 (Qiagen).

### 4.12 Hemin‐Agarose Pulldown Assays

Aliquots (~1 μg) of purified MBP‐Hhy1, MBP‐Hhy1MutA, MBP‐Hhy1MutB, and MBP proteins obtained from *E. coli* by amylose‐agarose chromatography were incubated with either hemin‐agarose or control agarose beads. The suspensions were mixed end‐over‐end for 30 min at 25°C. Beads were then centrifuged, and the unbound fractions were kept on ice. Subsequently, the beads were washed three times with phosphate‐buffered saline containing BSA (5 mg/mL). After the final wash, both the bead‐bound and unbound fractions were resuspended in SDS loading buffer (as described above) and heated for at 65°C for 30 min. Samples were resolved by electrophoresis on 8% SDS‐polyacrylamide gels and transferred to membranes for western blot analysis.

## Author Contributions

Conceptualization: Tobias Vahsen, Samuel Plante, Berthy Mbuya, and Simon Labbé. Methodology: Tobias Vahsen, Samuel Plante, Berthy Mbuya, and Simon Labbé. Validation: Tobias Vahsen, Samuel Plante, and Simon Labbé. Formal analysis: Tobias Vahsen, Samuel Plante, Berthy Mbuya, and Simon Labbé. Investigation: Tobias Vahsen, Samuel Plante, Berthy Mbuya, and Simon Labbé. Resources: Tobias Vahsen, Samuel Plante, Berthy Mbuya, and Simon Labbé. Writing – original draft preparation: Tobias Vahsen and Simon Labbé. Review and editing: Tobias Vahsen, Samuel Plante, Berthy Mbuya, and Simon Labbé. Supervision: Samuel Plante and Simon Labbé. Project administration: Simon Labbé. Funding acquisition: Simon Labbé. All authors have read, reviewed and approved the final version of the manuscript.

## Funding

This study was supported by the Natural Sciences and Engineering Research Council of Canada (NSERC, grant #RGPIN‐2020/2025‐04802) to S.L.

## Ethics Statement

The authors have nothing to report.

## Conflicts of Interest

The authors declare no conflicts of interest.

## Supporting information


**Figure S1:** jcmm71065‐sup‐0001‐FigureS1.pdf.

## Data Availability

All data are included in the present manuscript. Strains and plasmids used for this study are available from the corresponding author upon reasonable request. The authors state that all results obtained for confirming the conclusions presented in the article are represented fully within the article. Copyright statement: This is an open access article under the terms of the Creative Commons Attribution‐NonCommercial‐No Derivatives Licence (CC BY‐NC‐ND), which permits users to copy, distribute and transmit an article as long as the author is attributed. The article is not used for commercial purposes, and the work is not modified or adapted in any way.

## References

[mmi70062-bib-0001] Adam, M. , F. Robert , M. Larochelle , and L. Gaudreau . 2001. “H2A.Z Is Required for Global Chromatin Integrity and for Recruitment of RNA Polymerase II Under Specific Conditions.” Molecular and Cellular Biology 21: 6270–6279. 10.1128/MCB.21.18.6270-6279.2001. 11509669 PMC87352

[mmi70062-bib-0002] Andrawes, N. , Z. Weissman , M. Pinsky , S. Moshe , J. Berman , and D. Kornitzer . 2022. “Regulation of Heme Utilization and Homeostasis in *Candida albicans* .” PLoS Genetics 18: e1010390. 10.1371/journal.pgen.1010390. 36084128 PMC9491583

[mmi70062-bib-0003] Askwith, C. , and J. Kaplan . 1997. “An Oxidase‐Permease‐Based Iron Transport System in *Schizosaccharomyces pombe* and Its Expression in *Saccharomyces cerevisiae* .” Journal of Biological Chemistry 272: 401–405. 10.1074/jbc.272.1.401. 8995275

[mmi70062-bib-0004] Bairwa, G. , E. Sanchez‐Leon , E. Do , W. H. Jung , and J. W. Kronstad . 2020. “A Cytoplasmic Heme Sensor Illuminates the Impacts of Mitochondrial and Vacuolar Functions and Oxidative Stress on Heme‐Iron Homeostasis in *Cryptococcus neoformans* .” MBio 11: e00986. 10.1128/mBio.00986-20. 32723917 PMC7387795

[mmi70062-bib-0005] Baureder, M. , E. Barane , and L. Hederstedt . 2014. “In Vitro Assembly of Catalase.” Journal of Biological Chemistry 289: 28411–28420. 10.1074/jbc.M114.596148. 25148685 PMC4192493

[mmi70062-bib-0006] Brault, A. , B. Mbuya , and S. Labbé . 2022. “Sib1, Sib2, and Sib3 Proteins Are Required for Ferrichrome‐Mediated Cross‐Feeding Interaction Between *Schizosaccharomyces pombe* and *Saccharomyces cerevisiae* .” Frontiers in Microbiology 13: 962853. 10.3389/fmicb.2022.962853. 35928155 PMC9344042

[mmi70062-bib-0007] Brault, A. , C. Rallis , V. Normant , J. M. Garant , J. Bahler , and S. Labbé . 2016. “Php4 Is a Key Player for Iron Economy in Meiotic and Sporulating Cells.” G3: Genes, Genomes, Genetics 6: 3077–3095. 10.1534/g3.116.031898. 27466270 PMC5068932

[mmi70062-bib-0008] Chelikani, P. , I. Fita , and P. C. Loewen . 2004. “Diversity of Structures and Properties Among Catalases.” Cellular and Molecular Life Sciences 61: 192–208. 10.1007/s00018-003-3206-5. 14745498 PMC11138816

[mmi70062-bib-0009] Chen, D. , W. M. Toone , J. Mata , et al. 2003. “Global Transcriptional Responses of Fission Yeast to Environmental Stress.” Molecular Biology of the Cell 14: 214–229. 10.1091/mbc.E02-08-0499. 12529438 PMC140239

[mmi70062-bib-0010] Desuzinges‐Mandon, E. , O. Arnaud , L. Martinez , F. Huche , A. Di Pietro , and P. Falson . 2010. “ABCG2 Transports and Transfers Heme to Albumin Through Its Large Extracellular Loop.” Journal of Biological Chemistry 285: 33123–33133. 10.1074/jbc.M110.139170. 20705604 PMC2963377

[mmi70062-bib-0011] Donegan, R. K. , Y. Fu , J. Copeland , et al. 2022. “Exogenously Scavenged and Endogenously Synthesized Heme Are Differentially Utilized by *Mycobacterium tuberculosis* .” Microbiology Spectrum 10: e0360422. 10.1128/spectrum.03604-22. 36169423 PMC9604157

[mmi70062-bib-0012] Donegan, R. K. , C. M. Moore , D. A. Hanna , and A. R. Reddi . 2019. “Handling Heme: The Mechanisms Underlying the Movement of Heme Within and Between Cells.” Free Radical Biology & Medicine 133: 88–100. 10.1016/j.freeradbiomed.2018.08.005. 30092350 PMC6363905

[mmi70062-bib-0013] Dutt, S. , I. Hamza , and T. B. Bartnikas . 2022. “Molecular Mechanisms of Iron and Heme Metabolism.” Annual Review of Nutrition 42: 311–335. 10.1146/annurev-nutr-062320-112625. PMC9398995 35508203

[mmi70062-bib-0014] Engel, S. R. , S. Aleksander , R. S. Nash , et al. 2025. “Saccharomyces Genome Database: Advances in Genome Annotation, Expanded Biochemical Pathways, and Other Key Enhancements.” Genetics 229: yae185. 10.1093/genetics/iyae185. PMC11912841 39530598

[mmi70062-bib-0015] Hanna, D. A. , R. M. Harvey , O. Martinez‐Guzman , et al. 2016. “Heme Dynamics and Trafficking Factors Revealed by Genetically Encoded Fluorescent Heme Sensors.” Proceedings of the National Academy of Sciences of the United States of America 113: 7539–7544. 10.1073/pnas.1523802113. 27247412 PMC4941510

[mmi70062-bib-0016] Hanna, D. A. , R. Hu , H. Kim , O. Martinez‐Guzman , M. P. Torres , and A. R. Reddi . 2018. “Heme Bioavailability and Signaling in Response to Stress in Yeast Cells.” Journal of Biological Chemistry 293: 12378–12393. 10.1074/jbc.RA118.002125. 29921585 PMC6093230

[mmi70062-bib-0017] Hans, M. A. , E. Heinzle , and C. Wittmann . 2001. “Quantification of Intracellular Amino Acids in Batch Cultures of *Saccharomyces cerevisiae* .” Applied Microbiology and Biotechnology 56: 776–779. 10.1007/s002530100708. 11601629

[mmi70062-bib-0018] Ho, S. N. , H. D. Hunt , R. M. Horton , J. K. Pullen , and L. R. Pease . 1989. “Site‐Directed Mutagenesis by Overlap Extension Using the Polymerase Chain Reaction.” Gene 77: 51–59. 10.1016/0378-1119(89)90358-2. 2744487

[mmi70062-bib-0019] Igarashi, J. , M. Murase , A. Iizuka , F. Pichierri , M. Martinkova , and T. Shimizu . 2008. “Elucidation of the Heme Binding Site of Heme‐Regulated Eukaryotic Initiation Factor 2alpha Kinase and the Role of the Regulatory Motif in Heme Sensing by Spectroscopic and Catalytic Studies of Mutant Proteins.” Journal of Biological Chemistry 283: 18782–18791. 10.1016/0378-1119(89)90358-2. 18450746

[mmi70062-bib-0020] Ioannoni, R. , J. Beaudoin , A. Mercier , and S. Labbé . 2010. “Copper‐Dependent Trafficking of the Ctr4‐Ctr5 Copper Transporting Complex.” PLoS One 5: e11964. 10.1371/journal.pone.0011964. 20694150 PMC2915924

[mmi70062-bib-0021] Jacques, J. F. , A. Mercier , A. Brault , T. Mourer , and S. Labbé . 2014. “Fra2 Is a Co‐Regulator of Fep1 Inhibition in Response to Iron Starvation.” PLoS One 9: e98959. 10.1371/journal.pone.0098959. 24897379 PMC4045890

[mmi70062-bib-0022] Jbel, M. , A. Mercier , B. Pelletier , J. Beaudoin , and S. Labbé . 2009. “Iron Activates In Vivo DNA Binding of *Schizosaccharomyces pombe* Transcription Factor Fep1 Through Its Amino‐Terminal Region.” Eukaryotic Cell 8: 649–664. 10.1128/EC.00001-09. 19252122 PMC2669208

[mmi70062-bib-0023] Keeney, J. B. , and J. D. Boeke . 1994. “Efficient Targeted Integration at *leu1‐32* and *ura4‐294* in *Schizosaccharomyces pombe* .” Genetics 136: 849–856. 10.1093/genetics/136.3.849. 8005439 PMC1205890

[mmi70062-bib-0024] Koh, J. L. , Y. T. Chong , H. Friesen , et al. 2015. “CYCLoPs: A Comprehensive Database Constructed From Automated Analysis of Protein Abundance and Subcellular Localization Patterns in *Saccharomyces cerevisiae* .” G3: Genes, Genomes, Genetics 5: 1223–1232. 10.1534/g3.115.017830. 26048563 PMC4478550

[mmi70062-bib-0025] Kornitzer, D. , and U. Roy . 2020. “Pathways of Heme Utilization in Fungi.” Biochimica et Biophysica Acta, Molecular Cell Research 1867: 118817. 10.1016/j.bbamcr.2020.118817. 32777371

[mmi70062-bib-0026] Kuhl, T. , A. Wissbrock , N. Goradia , N. Sahoo , K. Galler , and U. Neugebauer . 2013. “Analysis of Fe(III) Heme Binding to Cysteine‐Containing Heme‐Regulatory Motifs in Proteins.” ACS Chemical Biology 8: 1785–1793. 10.1021/cb400317x. 23730736

[mmi70062-bib-0027] Kumar, S. , and U. Bandyopadhyay . 2005. “Free Heme Toxicity and Its Detoxification Systems in Human.” Toxicology Letters 157: 175–188. 10.1016/j.toxlet.2005.03.004. 15917143

[mmi70062-bib-0028] Labbé, S. , T. Mourer , A. Brault , and T. Vahsen . 2020. “Machinery for Fungal Heme Acquisition.” Current Genetics 66: 703–711. 10.1007/s00294-020-01067-x. 32185489

[mmi70062-bib-0029] Larochelle, M. , J. F. Lemay , and F. Bachand . 2012. “The THO Complex Cooperates With the Nuclear RNA Surveillance Machinery to Control Small Nucleolar RNA Expression.” Nucleic Acids Research 40: 10240–10253. 10.1093/nar/gks838. 22965128 PMC3488260

[mmi70062-bib-0030] Livak, K. J. , and T. D. Schmittgen . 2001. “Analysis of Relative Gene Expression Data Using Real‐Time Quantitative PCR and the 2(‐Delta Delta C(T)) Method.” Methods 25: 402–408. 10.1006/meth.2001.1262. 11846609

[mmi70062-bib-0031] Lorusso, C. , A. Calisi , G. Sarà , and F. Dondero . 2022. “In‐Gel Assay to Evaluate Antioxidant Enzyme Response to Silver Nitrate and Silver Nanoparticles in Marine Bivalve Tissues.” Applied Sciences 12: 2760. 10.3390/app12062760.

[mmi70062-bib-0032] Malcova, I. , M. Farkasovsky , L. Senohrabkova , P. Vasicova , and J. Hasek . 2016. “New Integrative Modules for Multicolor‐Protein Labeling and Live‐Cell Imaging in *Saccharomyces cerevisiae* .” FEMS Yeast Research 16: fow027. 10.1093/femsyr/fow027. 26994102

[mmi70062-bib-0033] Matsuyama, A. , R. Arai , Y. Yashiroda , A. Shirai , A. Kamata , and S. Sekido . 2006. “ORFeome Cloning and Global Analysis of Protein Localization in the Fission Yeast *Schizosaccharomyces pombe* .” Nature Biotechnology 24: 841–847. 10.1038/nbt1222. 16823372

[mmi70062-bib-0034] Mbuya, B. , S. Plante , F. Ammar , A. Brault , and S. Labbé . 2024. “The *Schizosaccharomyces pombe* Ornithine‐N(5)‐Oxygenase Sib2 Interacts With the N(5)‐Transacetylase Sib3 in the Ferrichrome Biosynthetic Pathway.” Frontiers in Microbiology 15: 1467397. 10.3389/fmicb.2024.1467397. 39328910 PMC11424930

[mmi70062-bib-0035] Mense, S. M. , and L. Zhang . 2006. “Heme: A Versatile Signaling Molecule Controlling the Activities of Diverse Regulators Ranging From Transcription Factors to MAP Kinases.” Cell Research 16: 681–692. 10.1038/sj.cr.7310086. 16894358

[mmi70062-bib-0036] Mercier, A. , and S. Labbé . 2009. “Both Php4 Function and Subcellular Localization Are Regulated by Iron via a Multistep Mechanism Involving the Glutaredoxin Grx4 and the Exportin Crm1.” Journal of Biological Chemistry 284: 20249–20262. 10.1074/jbc.M109.009563. 19502236 PMC2740451

[mmi70062-bib-0037] Mercier, A. , and S. Labbé . 2010. “Iron‐Dependent Remodeling of Fungal Metabolic Pathways Associated With Ferrichrome Biosynthesis.” Applied and Environmental Microbiology 76: 3806–3817. 10.1128/AEM.00659-10. 20435771 PMC2893484

[mmi70062-bib-0038] Mercier, A. , S. Watt , J. Bahler , and S. Labbé . 2008. “Key Function for the CCAAT‐Binding Factor Php4 to Regulate Gene Expression in Response to Iron Deficiency in Fission Yeast.” Eukaryotic Cell 7: 493–508. 10.1128/EC.00446-07. 18223116 PMC2268518

[mmi70062-bib-0039] Michener, J. K. , J. Nielsen , and C. D. Smolke . 2012. “Identification and Treatment of Heme Depletion Attributed to Overexpression of a Lineage of Evolved P450 Monooxygenases.” Proceedings of the National Academy of Sciences USA 109: 19504–19509. 10.1073/pnas.1212287109. PMC3511110 23129650

[mmi70062-bib-0040] Mourer, T. , A. Brault , and S. Labbé . 2019. “Heme Acquisition by Shu1 Requires Nbr1 and Proteins of the ESCRT Complex in *Schizosaccharomyces pombe* .” Molecular Microbiology 112: 1499–1518. 10.1111/mmi.14374. 31442344

[mmi70062-bib-0041] Mourer, T. , J. F. Jacques , A. Brault , M. Bisaillon , and S. Labbé . 2015. “Shu1 Is a Cell‐Surface Protein Involved in Iron Acquisition From Heme in *Schizosaccharomyces pombe* .” Journal of Biological Chemistry 290: 10176–10190. 10.1074/jbc.M115.642058. 25733668 PMC4400333

[mmi70062-bib-0042] Mourer, T. , V. Normant , and S. Labbé . 2017. “Heme Assimilation in *Schizosaccharomyces pombe* Requires Cell‐Surface‐Anchored Protein Shu1 and Vacuolar Transporter Abc3.” Journal of Biological Chemistry 292: 4898–4912. 10.1074/jbc.M117.776807. 28193844 PMC5377804

[mmi70062-bib-0043] Mutoh, N. , C. W. Nakagawa , and K. Yamada . 1999. “The Role of Catalase in Hydrogen Peroxide Resistance in Fission Yeast *Schizosaccharomyces pombe* .” Canadian Journal of Microbiology 45: 125–129. 10.1139/w98-216. 10380645

[mmi70062-bib-0044] Normant, V. , A. Brault , M. Avino , et al. 2021. “Hemeprotein Tpx1 Interacts With Cell‐Surface Heme Transporter Str3 in *Schizosaccharomyces pombe* .” Molecular Microbiology 115: 699–722. 10.1111/mmi.14638. 33140466

[mmi70062-bib-0045] Normant, V. , T. Mourer , and S. Labbé . 2018. “The Major Facilitator Transporter Str3 Is Required for Low‐Affinity Heme Acquisition in *Schizosaccharomyces pombe* .” Journal of Biological Chemistry 293: 6349–6362. 10.1074/jbc.RA118.002132. 29549126 PMC5925805

[mmi70062-bib-0046] Pelletier, B. , J. Beaudoin , Y. Mukai , and S. Labbé . 2002. “Fep1, an Iron Sensor Regulating Iron Transporter Gene Expression in *Schizosaccharomyces pombe* .” Journal of Biological Chemistry 277: 22950–22958. 10.1074/jbc.M202682200. 11956219

[mmi70062-bib-0047] Pelletier, B. , J. Beaudoin , C. C. Philpott , and S. Labbé . 2003. “Fep1 Represses Expression of the Fission Yeast *Schizosaccharomyces pombe* Siderophore‐Iron Transport System.” Nucleic Acids Research 31: 4332–4344. 10.1093/nar/gkg647. 12888492 PMC169938

[mmi70062-bib-0048] Pelletier, B. , A. Trott , K. A. Morano , and S. Labbé . 2005. “Functional Characterization of the Iron‐Regulatory Transcription Factor Fep1 From *Schizosaccharomyces pombe* .” Journal of Biological Chemistry 280: 25146–25161. 15866870 10.1074/jbc.M502947200

[mmi70062-bib-0049] Petrova, V. Y. , D. Drescher , A. V. Kujumdzieva , and M. J. Schmitt . 2004. “Dual Targeting of Yeast Catalase A to Peroxisomes and Mitochondria.” Biochemical Journal 380, no. Pt 2: 393–400. 10.1042/bj20040042. 14998369 PMC1224190

[mmi70062-bib-0050] Pezzoni, M. , R. A. Pizarro , and C. S. Costa . 2018. “Detection of Catalase Activity by Polyacrylamide Gel Electrophoresis (PAGE) in Cell Extracts From *Pseudomonas aeruginosa* .” Bio‐Protocol 8: e2869. 10.21769/BioProtoc.2869. 34285983 PMC8275273

[mmi70062-bib-0051] Ping, F. L. Y. , T. Vahsen , A. Brault , R. Néré , and S. Labbé . 2024. “The Flavohemoglobin Yhb1 Is a New Interacting Partner of the Heme Transporter Str3.” Molecular Microbiology 122: 29–49. 10.1111/mmi.15281. 38778742

[mmi70062-bib-0052] Plante, S. , A. Brault , M. Avino , et al. 2025. “The Heme‐Regulated Inhibitor Kinase Hri1 Is Activated in Response to Aminolevulinic Acid Deficiency in *Schizosaccharomyces pombe* .” PLoS Genetics 21: e1011797. 10.1371/journal.pgen.1011797. 40668850 PMC12303383

[mmi70062-bib-0053] Plante, S. , and S. Labbé . 2019. “Spore Germination Requires Ferrichrome Biosynthesis and the Siderophore Transporter Str1 in *Schizosaccharomyces pombe* .” Genetics 211: 893–911. 10.1534/genetics.118.301843. 30647069 PMC6404258

[mmi70062-bib-0054] Protacio, R. U. , T. O. Mukiza , M. K. Davidson , and W. P. Wahls . 2022. “Molecular Mechanisms for Environmentally Induced and Evolutionarily Rapid Redistribution (Plasticity) of Meiotic Recombination.” Genetics 220: iyab212. 10.1093/genetics/iyab212. 34888655 PMC9097252

[mmi70062-bib-0055] Reddi, A. R. , and I. Hamza . 2016. “Heme Mobilization in Animals: A Metallolipid's Journey.” Accounts of Chemical Research 49: 1104–1110. 10.1021/acs.accounts.5b00553. 27254265 PMC5629413

[mmi70062-bib-0056] Roy, U. , S. Yaish , Z. Weissman , et al. 2022. “Ferric Reductase‐Related Proteins Mediate Fungal Heme Acquisition.” eLife 11: e80604. 10.7554/eLife.80604. 36200752 PMC9635878

[mmi70062-bib-0057] Rustici, G. , H. van Bakel , D. H. Lackner , et al. 2007. “Global Transcriptional Responses of Fission and Budding Yeast to Changes in Copper and Iron Levels: A Comparative Study.” Genome Biology 8: R73. 10.1186/gb-2007-8-5-r73. 17477863 PMC1929147

[mmi70062-bib-0058] Rutherford, K. M. , M. Lera‐Ramírez , and V. Wood . 2024. “PomBase: A Global Core Biodata Resource‐Growth, Collaboration, and Sustainability.” Genetics 227: iyae007. 10.1093/genetics/iyae007. 38376816 PMC11075564

[mmi70062-bib-0059] Sabatinos, S. A. , and S. L. Forsburg . 2010. “Molecular Genetics of *Schizosaccharomyces pombe* .” Methods in Enzymology 470: 759–795. 10.1016/S0076-6879(10)70032-X. 20946835

[mmi70062-bib-0060] Sassa, S. 1976. “Sequential Induction of Heme Pathway Enzymes During Erythroid Differentiation of Mouse Friend Leukemia Virus‐Infected Cells.” Journal of Experimental Medicine 143: 305–315. 10.1084/jem.143.2.305. 1249519 PMC2190112

[mmi70062-bib-0061] Schmittgen, T. D. , and K. J. Livak . 2008. “Analyzing Real‐Time PCR Data by the Comparative C(T) Method.” Nature Protocols 3: 1101–1108. 10.1038/nprot.2008.73. 18546601

[mmi70062-bib-0062] Schrettl, M. , G. Winkelmann , and H. Haas . 2004. “Ferrichrome in *Schizosaccharomyces pombe*—An Iron Transport and Iron Storage Compound.” Biometals 17: 647–654. 10.1007/s10534-004-1230-z. 15689108

[mmi70062-bib-0063] Schubert, E. , N. Florin , F. Duthie , et al. 2015. “Spectroscopic Studies on Peptides and Proteins With Cysteine‐Containing Heme Regulatory Motifs (HRM).” Journal of Inorganic Biochemistry 148: 49–56. 10.1016/j.jinorgbio.2015.05.008. 26050879

[mmi70062-bib-0064] Severance, S. , and I. Hamza . 2009. “Trafficking of Heme and Porphyrins in Metazoa.” Chemical Reviews 109: 4596–4616. 10.1021/cr9001116. 19764719 PMC2769250

[mmi70062-bib-0065] Shimizu, T. , A. Lengalova , V. Martínek , and M. Martínková . 2019. “Heme: Emergent Roles of Heme in Signal Transduction, Functional Regulation and as Catalytic Centres.” Chemical Society Reviews 48: 5624–5657. 10.1039/c9cs00268e. 31748766

[mmi70062-bib-0066] Sweeny, E. A. , A. B. Singh , R. Chakravarti , et al. 2018. “Glyceraldehyde‐3‐Phosphate Dehydrogenase Is a Chaperone That Allocates Labile Heme in Cells.” Journal of Biological Chemistry 293: 14557–14568. 10.1074/jbc.RA118.004169. 30012884 PMC6139559

[mmi70062-bib-0067] Swenson, S. A. , C. M. Moore , J. R. Marcero , A. E. Medlock , A. R. Reddi , and O. Khalimonchuk . 2020. “From Synthesis to Utilization: The Ins and Outs of Mitochondrial Heme.” Cells 9: 579. 10.3390/cells9030579. 32121449 PMC7140478

[mmi70062-bib-0068] Vahsen, T. , A. Brault , T. Mourer , and S. Labbé . 2023. “A Novel Role of the Fission Yeast Sulfiredoxin Srx1 in Heme Acquisition.” Molecular Microbiology 120: 608–628. 10.1111/mmi.15146. 37644673

[mmi70062-bib-0069] Xue, P. , E. Sanchez‐Leon , D. Damoo , H. U. Guanggan , W. H. Jung , and J. W. Kronstad . 2022. “Heme Sensing and Trafficking in Fungi.” Fungal Biology Reviews 43: 100286. 10.1016/j.fbr.2022.09.002. 37781717 PMC10540271

