# Supplementary material for: The Hemoprotein Hhy1 Promotes Heme‐Dependent Catalase Activity of Ctt1
Source: Mol Microbiol. 2026 Mar 8;125(5):389–411. doi: 10.1111/mmi.70062 (PMC13135908; doi:10.1111/mmi.70062)
Supplement: Supplementary file 1 — Figure S1: jcmm71065‐sup‐0001‐FigureS1.pdf. [file MMI-125-389-s001.pdf]

Supplementary Information

The hemoprotein Hhy1 promotes heme-dependent catalase activity of Ctt1.

Tobias Vahsen, Samuel Plante, Berthy Mbuya, and Simon Labbé*.

Figure S1: Catalase activity is reduced in extracts from *hem1Δ hhy1Δ* cells compared with *hem1Δ* cells expressing the endogenous *hhy1*$^{+}$ gene.

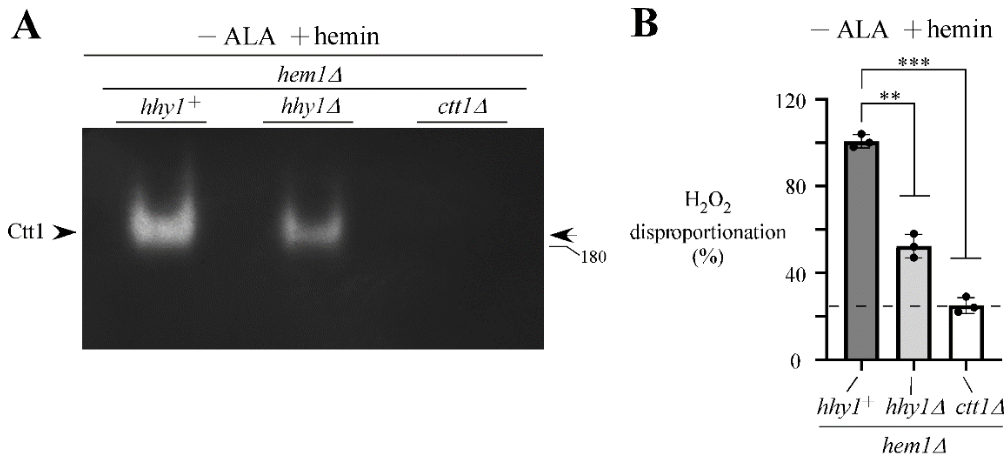

Fig. S1. Catalase activity is reduced in extracts from *hem1Δ hhy1Δ* cells compared with *hem1Δ* cells expressing the endogenous *hhy1*$^{+}$ gene.

A, The indicated strains were precultured in YES medium containing ALA (25 µM) and $FeCl_3$ (25 µM). At mid-logarithmic phase, the cultures were washed, resuspended in YES medium supplemented with Dip (25 µM), and then incubated without ALA for 6 h. Hemin (1 µM) was added to the cultures during the final 1.5 h of incubation. Total protein extracts from cultures were separated by non-denaturing polyacrylamide gel electrophoresis. Catalase activity was assessed and visualized by identifying H_2O_2-cleared bands following potassium ferricyanide/ferric chloride staining. *B*, Aliquots of cell lysates from *panel A* were analyzed spectrophotometrically at 240 nm to measure catalase activity. Activity was calculated based on the rate of H_2O_2 decomposition, proportional to the reduction in absorbance at 240 nm. Data are presented as mean ± SD. Statistical significance is indicated by asterisks: $^{**}p < 0.01$ and $^{***}p < 0.001$ (determined by one-way ANOVA with Dunnett's multiple comparisons test, using *hem1*Δ cells expressing endogenous *hhy1*$^{+}$ as the reference).
